# Dicobalt(ii) helices kill colon cancer cells *via* enantiomer-specific mechanisms; DNA damage or microtubule disruption†

**DOI:** 10.1039/d4sc02541e

**Published:** 2024-06-14

**Authors:** Hualong Song, Hana Kostrhunova, Jakub Cervinka, Julie Macpherson, Jaroslav Malina, Teena Rajan, Roger Phillips, Miles Postings, Samantha Shepherd, Xuejian Zhang, Viktor Brabec, Nicola J. Rogers, Peter Scott

**Affiliations:** a Beijing Area Major Laboratory of Peptide and Small Molecular Drugs, Engineering Research Centre of Endogenous Prophylactic of Ministry of Education of China, School of Pharmaceutical Sciences, Capital Medical University Beijing 100069 China hualong.song@ccmu.edu.cn; b Department of Chemistry, University of Warwick Coventry CV4 7AL UK peter.scott@warwick.ac.uk; c Czech Academy of Sciences, Institute of Biophysics Brno Czech Republic brabec@ibp.cz; d Faculty of Science, Department of Biochemistry, Masaryk University Brno Czech Republic; e Department of Pharmacy, University of Huddersfield Huddersfield HD1 3DH UK; f College of Chemistry, State Key Laboratory of Elemento-Organic Chemistry, Nankai University Tianjin 300071 China; g Department of Biophysics, Palacky University Olomouc Czech Republic; h Department of Chemistry, Hong Kong Baptist University Kowloon Tong Hong Kong SAR China nicolarogers@hkbu.edu.hk

## Abstract

Highly diastereoselective self-assembly reactions give both enantiomers (Λ and Δ) of anti-parallel triple-stranded bimetallic Co(ii) and Co(iii) cationic helices, without the need for resolution; the first such reaction for Co. The complexes are water soluble and stable, even in the case of Co(ii). Studies in a range of cancer and healthy cell lines indicate high activity and selectivity, and substantial differences between enantiomers. The oxidation state has little effect, and correspondingly, Co(iii) compounds are reduced to Co(ii) *e.g.* by glutathione. In HCT116 colon cancer cells the Λ enantiomer induces dose-dependent G2-M arrest in the cell cycle and disrupts microtubule architectures. This Co(ii) Λ enantiomer is *ca.* five times more potent than the isostructural Fe(ii) compound. Since the measured cellular uptakes are similar this implies a higher affinity of the Co system for the intracellular target(s); while the two systems are isostructural they have substantially different charge distributions as shown by calculated hydrophobicity maps. In contrast to the Λ enantiomer, Δ-Co(ii) induces G1 arrest in HCT116 cells, efficiently inhibits the topoisomerase I-catalyzed relaxation of supercoiled plasmid DNA, and, unlike the isostructural Fe(ii) system, causes DNA damage. It thus seems very likely that redox chemistry plays a role in the latter.

## Abstract 2

Optically pure Co metallohelices exhibit high and selective potency against cancer cells but operate by different mechanisms; the Δ-enantiomer induces DNA damage in HCT116 cells while the Λ-enantiomer disrupts tubulin networks.

## Graphical abstract

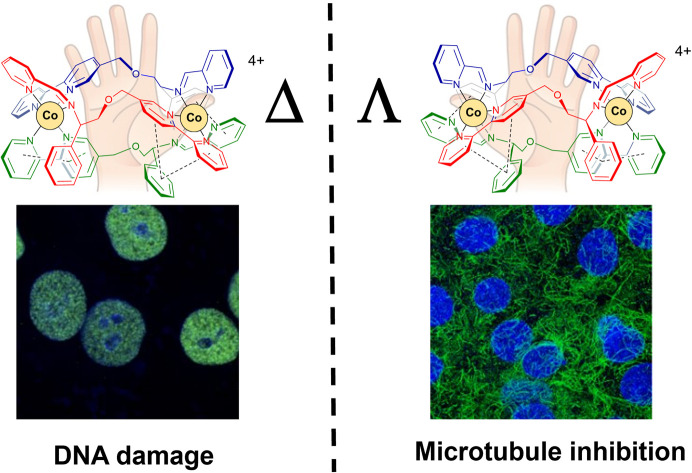

## Introduction

We have reported several classes of optically pure helical Fe(ii) and Zn(ii) assemblies,^1–7^ in which, unlike conventional helicates^8–12^ the sense of helicity in the assembly (Δ or Λ) is fixed in highly diastereoselective processes at individual metal centres.^13^ The Fe(ii) compounds have unexpectedly high stability to hydrolysis, despite the conventional lability of this ion; we found that this arises from a combination of hydrophobic π-stacking, hydrogen bonding and in some cases mechanical coupling between the metal coordination spheres. This unique combination of essentially perfect stereocontrol and high stability in water has enabled extensive studies by various teams in the areas of cancer,^2,4,5,14–16^ (notably binding to G-quadruplexes and other DNA features,^17–27^) antimicrobials,^1,7^ Alzheimer's disease,^28–30^ diabetes,^31^ gene delivery,^22^ and inhibition of ice recrystallisation.^32^ Overall this has led to the observation that the compounds emulate the properties short cationic α-helical peptides.^33^

We noted recent elegant syntheses of racemic Co(iii) supramolecular architectures *via* oxidation of the labile Co(ii) compounds,^34–37^ and set out to see if optically pure, water-stable metallohelices could be accessed so that the biological effects, particularly in cancer cells, could be compared with the parent Fe systems.

## Results and discussion

### Self-assembly of stable optically pure Co triplexes

**Scheme 1:**
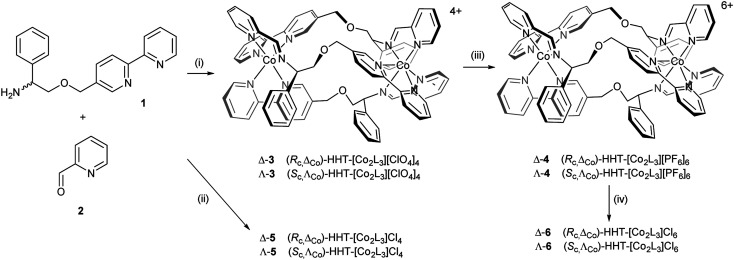
Synthesis of cobalt(ii) and cobalt(iii) metallohelices with various counter-ions: (i) Co(ClO_4_)_2_·6H_2_O; (ii) CoCl_2_; (iii) (NH_4_)_2_[Ce(NO_3_)_6_], NH_4_PF_6_; (iv) Amberlite® IRA-400 (chloride form) resin.

Cobalt(ii) perchlorate hexahydrate and appropriate proportions of single enantiomers of 2-([2,2′-bipyridin]-5-ylmethoxy)-1-phenylethan-1-amine (*R*-1 and *S*-1, 3 equiv.)^4^ and 2-pyridinecarboxaldehyde 2 self-assembled on heating to give bright yellow optically pure Co(ii) compounds (*S*_c_,Λ_Co_)- and (*R*_c_,Δ_Co_)-HHT-[Co_2_L_3_][ClO_4_]_4_ (3) (Scheme 1). The ^1^H NMR spectra (ESI, Fig. S1†) show the presence single diastereomeric species *i.e.* essentially a single enantiomer, and also that one ligand strand is oriented in the opposite sense to the other two *i.e.* the so-called head-head-tail (HHT) or triplex structure.^4^ We know of no other self-assembled optically pure Co structures of this kind, although of course the chemical resolution of (inert) Co(iii) compounds led to the dawn of synthetic coordination chemistry.^38^

The Co(ii) metallohelices 3 were cleanly oxidized to Co(iii) with ceric ammonium nitrate^34–37^ and isolated as the orange crystalline hexafluorophosphate salts Δ- and Λ-4. NMR spectra of diamagnetic enantiomers 4 (Fig. S3 and S4†) indicates that the antiparallel structure has been preserved during oxidation.

Water soluble cobalt(ii) triplex metallohelix enantiomers (*R*_c_,Δ_Co_)- and (*S*_c_,Λ_Co_)-[Co_2_L_3_]Cl_4_, Λ- and Δ-5 were prepared by self-assembly of ligand precursors with cobalt(ii) chloride, and as above, the ^1^H NMR spectra exhibited large hyperfine shifts (Fig. S2†). The average *μ*_eff_ of each Co(ii) centre of Δ-5 was estimated by the Evans method^39^ in D_2_O as 4.3 ± 0.1 BM, assuming independent spins, and this is reasonable given a spin-only *μ*_eff_ of 3.9 BM for high spin octahedral Co(ii), considerable orbital contribution and possible spin–orbit coupling.^40^ The water soluble Co(iii) metallohelices (*R*_c_,Δ_Co_)- and (*S*_c_,Λ_Co_)-[Co_2_L_3_]Cl_6_ (Δ- and Λ-6) were prepared conveniently by anion exchange of 4 using Amberlite® IRA-400 (chloride form) resin. The ^1^H NMR spectra in D_2_O (Fig. S3 and S5†) were similar in most respects to those of the hexafluorophosphates in CD_3_CN. Five singlets (9.9–9.3 ppm) are assigned to the three imine and two of the bpy H atoms (H_a_ and H_b_), with H_c_ appearing at 7.6 ppm.

**Fig. 1:**
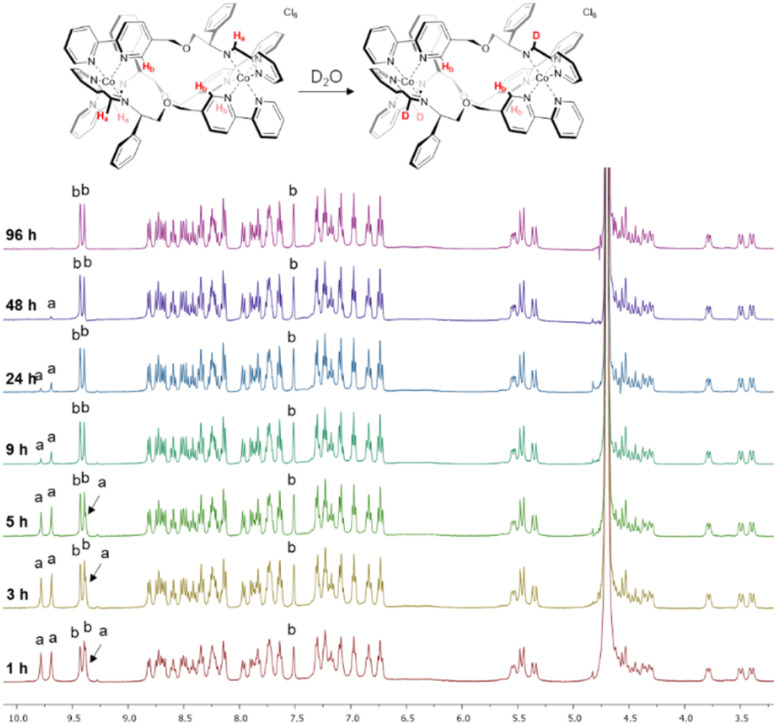
NMR deuterium exchange in cobalt(iii) metallohelices. The imine deuterium exchange of Λ-6 monitored by ^1^H NMR spectroscopy (400 MHz, 298 K, D_2_O).

We observed however that the three imine protons H_a_ slowly disappear from the spectrum over 48 h (Fig. 1) due to deuterium exchange in D_2_O as confirmed by mass spectrometry (Fig. S11†). Such behaviour is not observed in the divalent metal complexes of Co above, Zn, or Fe,^4^ and is attributed here to the greater acidity of the imine protons when coordinated to a tricationic metal ion. More detailed stability studies are described later.

### Co(ii) systems have amphipathic architectures

To probe the effects of changing the identity and oxidation state of the metal centres on charge distribution, structures of the Δ-metallohelix cations of 5, 6 and the Fe(ii)-analogue ‘Δ-Fe^II^’, were calculated using DFT methods (see ESI Section 5†). The computationally-predicted structures have average M–N bond lengths of 1.97, 1.94, and 1.93 Å for the Co(ii), Fe(ii), and Co(iii) triplex metallohelices respectively, which compares to average M–N bond lengths of 2.16, 1.97, and 1.94 Å respectively^13^ from experimental crystal structures of monometallic phenylethaniminopyridine tris-chelates.

**Fig. 2:**
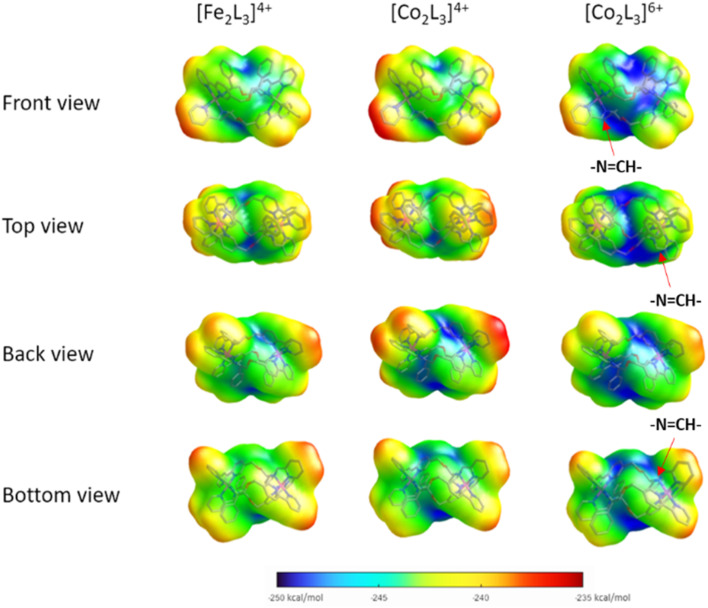
Hydrophobicity maps of metallohelices. Views of DFT-calculated structures and overlayed hydrophobicity plots (binding energy of individual water molecules, plotted onto the surface of the optimised geometry) for (*R*_c_,Δ_Fe_)-[Fe_2_L_3_]^4+^ (Δ-Fe^II^), (*R*_c_,Δ_Co_)-[Co_2_L_3_]^4+^ (Δ-5), and (*R*_c_,Δ_Co_)-[Co_2_L_3_]^6+^ (Δ-6).

The helical folding of the three antiparallel strands in the triplex structures 3–6, as well as their Fe(ii) analogues, creates asymmetric shielding of the cationic charge arising from the metal centres, leading to amphipathic architectures. Hydrophobicity maps,^32^ based on the single point energies of a water molecules at multiple optimised positions on the surface of the metallohelix (Fig. 2) indicate distinct hydrophobic regions, mainly corresponding to the external faces of the π-stacked phenyl rings, and more hydrophilic regions *e.g.* near the ether bridges and exposed elements of coordination sphere. As expected, the Co(iii) metallohelix is substantially more hydrophilic than both the Co(ii) and Fe(ii) analogues. Most importantly in respect of the biological activity presented below, there are also some quite striking differences between the d^6^ Fe(ii) and d^7^ Co(ii) architectures, with the latter being more amphipathic in nature *i.e.* with more distinct patches, particularly the back face (Fig. 2); the π-stacked arenes are more hydrophobic and the centre of the architecture is more hydrophilic in the Co(ii) structure than its Fe(ii) analogue.

Notably, the three imine protons of the Co(iii) complex 6 are localized in distinctly hydrophilic regions (more positively charged) compared with the Fe(ii) and Co(ii) (5) complexes, correlating with the deuterium exchange observed in D_2_O solutions of 6 only. Due to the asymmetric HHT geometry of the triplex metallohelices, the hydrophilicity of each imine proton environment is also slightly different, consistent with the differing deuterium exchange rates observed (Fig. 1).

### Metallohelices principally exist in Co(ii) form *in cellulo*

Cyclic voltammetry experiments were carried out in water (Fig. S17†). Λ-5 exhibited a single oxidation peak, with a reduction potential of +189 mV *vs.* a standard calomel electrode (*i.e.* +436 mV *vs.* NHE). The rather high peak-to-peak separation of 93 mV probably results from the overlap of two separate redox potentials for the metal centres which have sufficiently different ligation.^41^ We also note that slow electron transfer (associated with quasi-reversible behaviour) is expected given the change in spin multiplicity [*i.e.* high spin Co(ii) to low spin Co(iii)].^42^

**Fig. 3:**
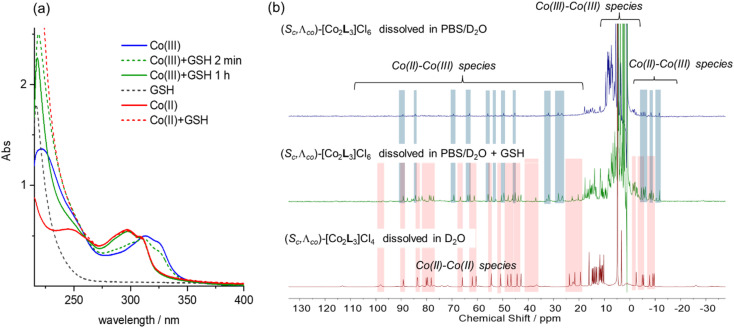
Reduction of Co(iii) systems with glutathione: (a) UV-vis spectra of Λ-6 (10 μM) in H_2_O (solid blue line); Λ-6 in GSH (1 mM) and pH 7.4 Tris buffer at 2 min (green dotted line), and at 1 h (green solid line); GSH (1 mM) in pH 7.4 Tris buffer (grey dotted line); Λ-5 (10 μM) in H_2_O (red solid line); Λ-5 in GSH (1 mM)/pH 7.4 Tris buffer (red dotted line); (b) ^1^H NMR spectra (300 MHz, 298 K) of Λ-6 (2 mM) in PBS/D_2_O (10 mM, 0.14 M NaCl, pD = 7.4) (top), Λ-6 (2 mM) in PBS/D_2_O (10 mM, 0.14 M NaCl, pD = 7.4) plus 10 mM GSH (middle), and Λ-5 (2 mM) in PBS/D_2_O (10 mM, 0.14 M NaCl, pD = 7.4) (bottom). All samples were spiked with ^*t*^BuOH (5 mM), and signal intensities normalized to ^*t*^BuOH.

In eukaryotic cells, the redox potential is controlled between *ca.* −450 and −150 mV *vs.* NHE and compartmentalized, and the extracellular potential in culture medium is regulated to be similar that in plasma.^43^ In this potential range we would expect the Co metallohelices to exist in the divalent state *i.e.* that the Co(iii) metallohelices 6 would be reduced in either cell media or cytoplasm. While reactive oxygen species, thioredoxin, NADH, and NADPH are involved in modulation of cellular potential, the predominant redox buffer is considered to be glutathione (GSH), which is found in millimolar (5–10 mM) concentrations in cells.^44^ We thus monitored the UV-vis absorption spectra of the Co metallohelices in the presence of glutathione [Fig. 3(a)]. Addition of 1 mM GSH to a 10 μM stock solution of the Co(iii) complex Λ-6 was followed by a gradual hypsochromic shift over 1 h, and the final spectrum (green line) superimposed that of Co(ii) complex Λ-5 at same concentration (red line), indicating quantitative reduction.

We also monitored the effect of glutathione on the Co(iii) metallohelices by ^1^H NMR spectroscopy. A PBS/D_2_O solution of Λ-6 (2 mM) showed peaks outside of the 0–10 ppm diamagnetic range [Fig. 3(b), *top*] consistent with the presence of a small amount of a Co(iii)/(ii) mixed-valence species in that the spectrum did not correspond to authentic Co(ii) compound Λ-5 and there were *ca.* half the number of expected peaks. We would expect the Co metal centre coordinated by two imine-pyridyl ligands to be first reduced to Co(ii).^41^ The addition of 10 mM GSH into a similar 2 mM solution of Λ-6 (spiked with 5 mM *tert*-butanol as internal marker, 10 mM PBS) caused the decrease of Co(iii)–Co(iii) complex peaks by factor of 2.4 (Fig. 3(b)*middle* and Fig. S19†) whilst new protons observed, which were assigned to Co(ii)–Co(ii) complex [by comparison to the spectrum of complex Λ-5, Fig. 3(b)*bottom*], and a smaller residual set of Co(iii)–Co(ii) metallohelix peaks (approximately 50% intensity of Co(ii)–Co(ii) resonances) are also observed.

In addition to long-term stability studies by ^1^H NMR in D_2_O, which showed no decomposition for 3 weeks or more (Fig. S12†), solutions of cobalt(ii) and cobalt(iii) triplexes were monitored over time by UV/vis spectroscopy in PBS and cell media (Fig. S13†). In the latter the Co(iii) systems were, as now expected, reduced cleanly to Co(ii), and otherwise the systems showed excellent stability.

### Λ enantiomers are more active against cancer cells, independent of oxidation state

The water-soluble triplex metallohelices 5 and 6 were screened for potency against a panel of six cancer cell lines, plus the non-cancerous MRC5 (Table 1). Very similar results were found in another of our laboratories (Table S2†). A striking enantioselectivity was observed, with the cytotoxic/antiproliferative effect of the Λ enantiomers consistently higher than that of Δ enantiomers. Also, negligible or much smaller differences were observed between Co(ii) and Co(iii) compounds, consistent with reduction of Co(iii) in the cells and/or media *i.e.* the active species being Co(ii), as expected from electrochemical studies above. The compounds are significantly less toxic to the non-cancerous MRC5 (Table 1) and ARPE-19 (Table S2†) cell lines. Further, the activity of the Co compounds is three- to ten-fold higher than the isostructural Fe complexes (Table S2†) pointing to an effect of the difference in charge density/hydrophobicity between the two divalent series, and/or an electrochemical effect. We also note that compounds Λ-5/6 are *ca.* ten times more active than the clinical chemotherapeutic drug cisplatin.^14^

**Table 1:** Cell viability (IC_50_) determined *via* MTT assay. Experiments were performed in triplicate, cells were incubated with compounds for 72 h, results are expressed as MEAN ± SD

	Metal ion	IC_50_ value (μM) ± SD						
		HCT116	Colo320	MCF7	RD	HeLa	PSN1	MRC5
Λ-5	Co(ii)	0.7 ± 0.1	0.8 ± 0.1	0.70 ± 0.09	0.67 ± 0.09	0.9 ± 0.1	0.6 ± 0.1	3.9 ± 0.8
Δ-5	Co(ii)	3.5 ± 0.7	2.2 ± 0.2	3.4 ± 0.8	2.0 ± 0.5	2.4 ± 0.5	2.1 ± 0.3	13 ± 2
Λ-6	Co(iii)	0.8 ± 0.2	0.94 ± 0.06	1.0 ± 0.3	0.7 ± 0.1	1.0 ± 0.2	0.82 ± 0.09	5 ± 1
Δ-6	Co(iii)	6 ± 1	7 ± 1	4.9 ± 0.8	5.7 ± 0.9	7 ± 1	4.4 ± 0.7	21 ± 5

### Co(ii) enantiomers achieve higher cellular and nuclear concentration

HCT116 cells were exposed to the four Co compounds at 5 μM for 8 h (*cf.* 72 or 96 h exposure for the MTT assays above). The intracellular and nuclear amounts of cobalt were determined by ICP MS following cell lysis or using a commercial kit respectively and expressed as ng Co per 10^6^ cells/nuclei (Table 2). Notably, while the whole cell accumulations of Co(ii) metallohelices at this short exposure time were similar to those measured in the isostructural Fe(ii) systems studied in detail recently in the same cell line,^16^ and the enantiomeric differences were similarly insignificant, we see that the Co(iii) compounds achieve *ca.* 50% lower accumulation. This suggests, perhaps unsurprisingly, that the less lipophilic Co(iii) compounds (6+ charge overall) have slower intracellular transport, and also that the majority of reduction to Co(ii) is happening *in cellulo*. The nuclear accumulations are roughly proportional to the respective cellular concentrations.

**Table 2:** Cellular and nuclear accumulation. Concentration of the metallohelices in HCT116 cells following 8 h exposure to the compounds (5 μM). Nuclei were isolated with Nuclei EZ Prep Kit. Table shows MEAN ± SD from three independent measurements

	ng Co/10^6^ cells	ng Co/10^6^ nuclei
Λ-5	29 ± 3	1.7 ± 0.2
Δ-5	33 ± 4	2.1 ± 0.2
Λ-6	17 ± 2	0.8 ± 0.2
Δ-6	16 ± 3	1.0 ± 0.3

### Co(ii) compounds favour programmed cell death

Apoptosis is a form of highly regulated and controlled *i.e.* programmed cell death, proceeding *via* cell morphology changes. In contrast, necrosis results from acute cellular injury. HCT116 cells were treated with metallohelices at concentrations corresponding to 3 × IC_50_ values for 48 h, and apoptotic and necrotic population percentages were recorded using annexin V/propidium iodide staining. Staurosporine and ethanol treated samples were added as apoptotic and necrotic inducer controls respectively (Fig. S20†). Notably, a higher proportion of apoptosis was observed with the less highly charged Co(ii) compounds.

### Metallohelix enantiomers kill cancer cells by different mechanisms

**Fig. 4:**
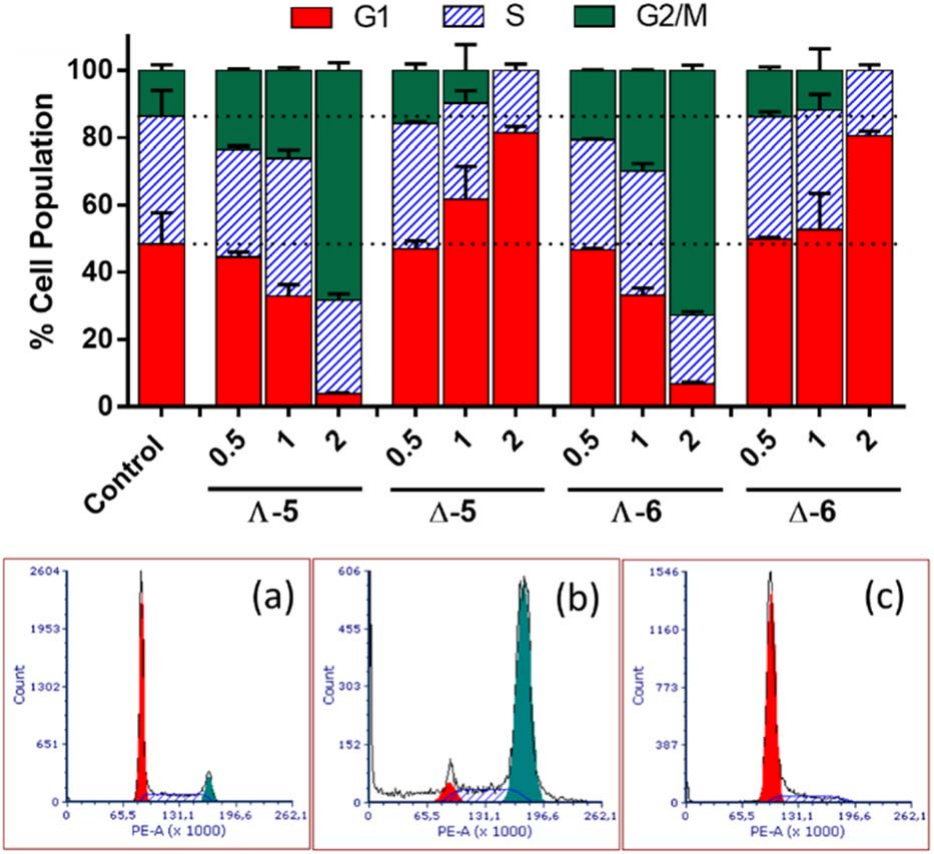
Cell cycle distribution of HCT116 cells following a 24 h treatment with cobalt metallohelices Λ-5, Δ-5, Λ-6, and Δ-6; control – untreated cells; 0.5, 1, and 2 – the cells were treated at concentrations corresponding to 0.5×, 1× and 2 × IC_50_ (MTT; 72 h), respectively; (a–c) show representative cell cycle profiles of (a) untreated HCT116 cells, or cells treated with (b) Λ-5 and (c) Δ-5. Cells were assessed with FACS following staining with propidium iodide.

In cell cycle studies, HCT116 cells were exposed to the new compounds for 24 h at increasing multiples of IC_50_ (Fig. 4). Co(ii) and Co(iii) compounds of the same structure behaved similarly, but Λ and Δ isomers affected the cell cycle very differently.

The Λ enantiomers induced significant and dose dependent increase in G2/M population at the expense of G1, such that even at 0.5 × IC_50_*i.e.* 350 nM, changes in cell cycle profiles are clear, and at 2 × IC_50_ (1.4 μM) less than 10% of cells were in G1. For the isostructural Fe(ii) compound, a very similar cell cycle arrest picture is seen, albeit at much higher concentration *ca.* 7 × IC_50_ (10 μM), otherwise under the same conditions.^16^ Thus, the higher potency of Λ-Co^II^ (5) *versus* Λ-Fe^II^ is reflected in the cell cycle behaviour. Cells in the G2 and mitotic phases are detected together by flow cytometry as a G2/M population, so Λ-5 could be causing cell cycle arrest at: (i) the G2-M checkpoint, which occurs after the G2 phase (rapid cell growth, protein synthesis and preparation for mitosis), perhaps as a result of the Λ-metallohelix binding to DNA, or (ii) the spindle assembly checkpoint (SAC) which occurs during mitosis and prevents progression to the anaphase, and probably resulting from interference with *e.g.*, the function of cytoskeleton proteins.

The Δ enantiomers had highly contrasting although still dose-dependent behaviour (Fig. 4), arresting the cells in G1 with the remaining population in S phase, such that at 2 × IC_50_ (7 μM) there has been a 60% increase in the proportion of cells in G1 (*ca.* 50% to 80%) and negligible G2/M population remaining. This effect is far greater than that which we observed^16^ for the Fe analogue Δ-Fe^II^; a 25% increase in G1 (40% to 50%) at 2 × IC_50_, noting that this corresponds to 40 μM given the rather lower potency of that compound.

During the G1 phase, the cell undergoes growth and synthesizes the material needed for DNA synthesis in the S phase. At the G1/S checkpoint a cell may be cleared for progression to this synthesis phase, or if it is signalled to remain undivided it will leave the cell cycle and become dormant (G0 phase). Alternatively, if there has been insufficient growth *e.g.* because of lack of nutrients, or there is damaged DNA, the cell may remain (be arrested) in G1.^45^ The behaviour of Δ-Fe^II^ was similar,^16^ although a less significant increase in the proportion of cells in G1 phase was observed at 2 × IC_50_ (only a 25% increase).

Due to the marked differences in the effects of the enantiomers on cell cycle progression, we also employed real time impedance-based monitoring of cell growth, which monitors the impedance of cells,^14,46^ and compared the response profiles of HCT116 cells treated with Λ/Δ-5 and Λ/Δ-6 (Fig. S21†). Similar to the cell-cycle data, the time-dependent cellular response profiles (TCRPs) of HCT116 cells reveal clear differences between the Λ and Δ enantiomers, so that while the TCRPs of cells treated with Λ-5 or 6 trace that of the control cells for 24 h and then drop in a concentration dependent manner, those of the cells treated with Δ-enantiomers induce an initial increase in the impedance signal followed by a delayed concentration dependent decrease. The different TCRPs between the enantiomers reflect different adhesion, growth and/or morphology of the cells, indicative of different mechanisms of action.

These distinct observations on cell cycle effects and cell impedance for the two enantiomers led us to investigate their effects on DNA processes/damage and cytoskeleton proteins.

### Metallohelix enantiomers have distinct effects on DNA processing and damage

**Fig. 5:**
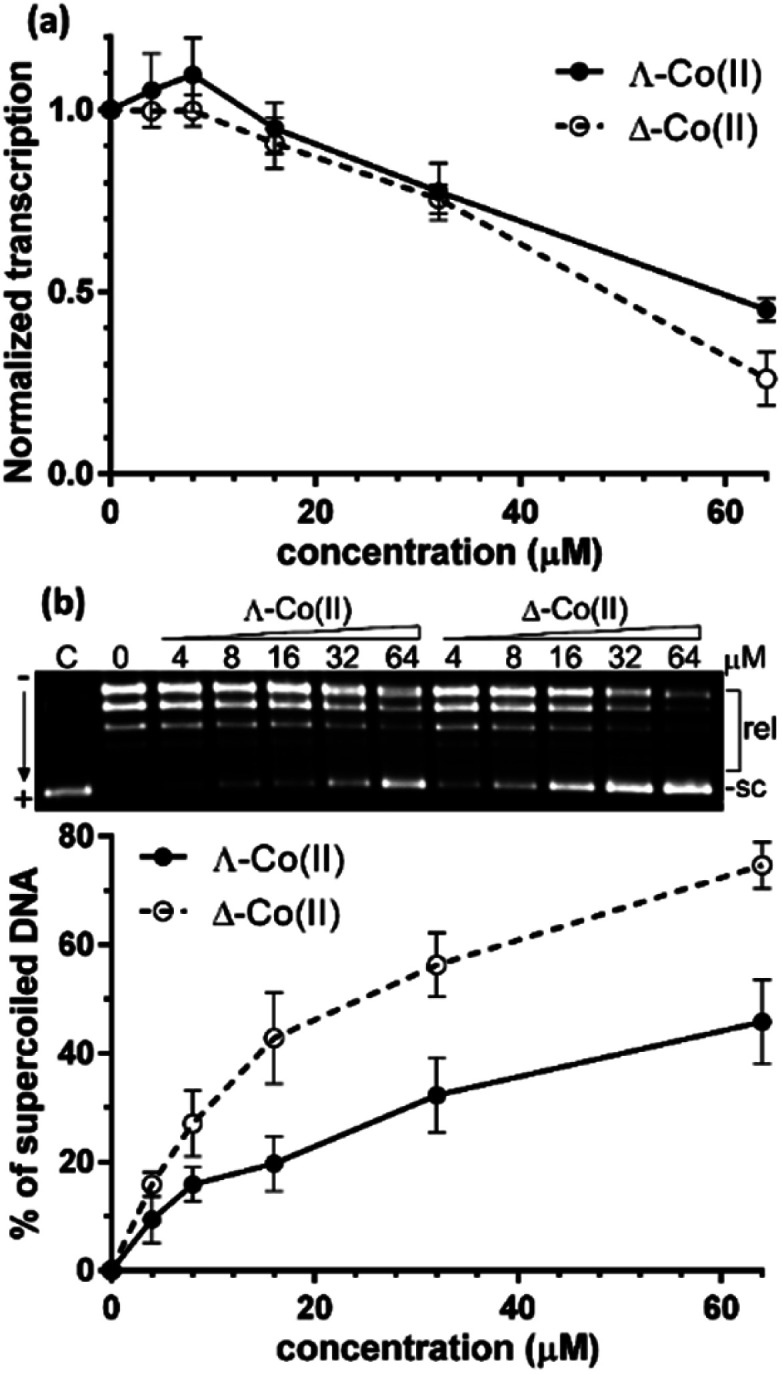
Inhibition of DNA transcription: (a) plot of the relative transcriptional activity of pBR322 plasmid DNA *vs.* the concentration of Co(ii) metallohelices. The concentration of DNA was 30 μM (per nucleotide); (b) inhibition of relaxation of negatively supercoiled pUC19 plasmid DNA by topoisomerase I in the presence of increasing concentrations (indicated above the gel) of Co(ii) metallohelices. The concentration of DNA in the samples was 78 μM (per nucleotide). *sc* and *rel* correspond to supercoiled and relaxed forms of plasmid DNA, respectively. Plot shows the % of supercoiled form of plasmid DNA as a function of concentration of metallohelices.

We first sought to assess the ability of enantiomers 5 to inhibit the transcription of DNA. The assay^47^ (see ESI†) uses a circular plasmid DNA as the template and a fluorescent analogue of uridine triphosphate as one of the nucleotide substrates. Incorporation of the uridine into an RNA strand by RNA polymerase leads to the release of the fluorescent tag. Transcription was inhibited by the presence of increasing concentrations of either enantiomer [Fig. 5(a)] with Δ-5 being slightly the more efficient at higher concentrations.

Greater enantioselectivity was observed in inhibition of topoisomerase I-catalysed relaxation of negatively supercoiled DNA. Topoisomerases are essential enzymes involved in the regulation of DNA supercoiling and participate in nearly all events related to DNA metabolism. The conversion of naturally negatively supercoiled pUC19 plasmid in the presence of increasing concentrations of Co(ii) enantiomers 5 into relaxed covalently closed circular DNA was monitored by using gel electrophoresis [Fig. 5(b)]. Here, the Δ-5 was significantly the more potent inhibitor.

**Fig. 6:**
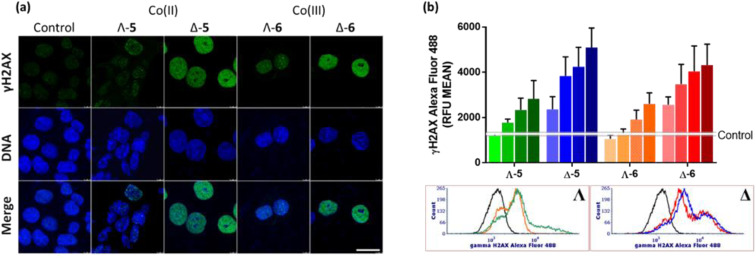
Phosphorylation of H2AX. (a) Confocal fluorescence images: HCT116 cells were non-treated or treated with the metallohelices at 4 μM concentration for 24 h. Cell nuclei were stained with DAPI (DNA) γH2AX was detected with anti-γH2AX antibody and secondary antibody conjugated with Alexa Fluor 488. Scale bar represents 20 μm. (b) Flow cytometry analysis of H2AX phosphorylation in HCT116 cells: treated with the cobalt metallohelices at 0.5 μM, 1 μM, 2 μM and 4 μM concentrations for 24 h, then immunostained with Alexa Fluor® 488 conjugated anti γH2AX antibody and analysed with FACS Verse flow cytometer (3 × 10^4^ cells per sample). The data were obtained using ModFit software and are shown as MEAN ± SD of two independent experiments. Representative histograms of cells treated with 4 μM concentrations are shown: black – non-treated cells, green – Co(ii)-Λ, orange – Co(iii)-Λ, blue – Co(ii)-Δ, red – Co(iii)-Δ.

We have also investigated γH2AX induction in HCT116 cells exposed to the Co compounds. Phosphorylation of the histone protein H2AX at serine 139 to produce γH2AX is a known cellular response to double strand breaks,^48^ when progression of DNA replication is halted^49^*e.g.* by malfunctioning DNA or RNA polymerase complexes^50^ or DNA damage caused by drugs.^51,52^ γH2AX has been detected as discrete foci, correlating to the number of double strand breaks,^53^ or as pan-nuclear H2AX phosphorylation.^54^Fig. 6(a) shows representative confocal images of γH2AX presence in cellular nuclei, detected *via* green fluorescent antibodies. In untreated control cells, the green fluorescence is negligible, and only modest H2AX phosphorylation is observed for cells treated with Λ-5/6; weak green fluorescence is observed throughout the nuclei, with a small number of distinguishable foci, despite the compound concentration corresponding to *ca.* 5 × IC_50_. In contrast, treatment of the cells with either Δ-5 or Δ-6 at the same concentration (*ca.* 1 × IC_50_) triggered strong widespread H2AX phosphorylation, indicating DNA damage. It is noteworthy that neither of the isostructural Fe(ii) enantiomers caused DNA damage as indicated by γH2AX assay.^4^

The results from confocal microscopy were confirmed and quantified using flow cytometry of HCT116 cells treated with enantiomers 5 and 6 at equimolar concentrations [Fig. 6(b)]. While the Λ compounds induced H2AX phosphorylation at higher concentrations, Δ enantiomers led to pronounced response even at 0.5 μM, despite these being substantially less potent antiproliferative agents (Tables 1 and S2†). Representative flow cytometry histograms are shown [Fig. 6(b)*bottom*].

### Λ enantiomer strongly inhibits tubulin polymerization

Due to the observation of accumulation of HCT116 cells in the G2/M phase upon treatment with Λ-5 (Fig. 4), we investigated the effect of enantiomers 5 on actin and tubulin. Many agents that induce accumulation in G2/M act *via* disturbing microtubule dynamics through inhibition of tubulin polymerization (*e.g.* vinca alkaloids which block beta-tubulin polymerization and thus prevent cellular division^55,56^) or stabilization of microtubules (*e.g.* paclitaxel which disturbs mitotic spindle assembly and chromosome segregation thus blocking the progression of mitosis, and triggers apoptosis).^57^

**Fig. 7:**
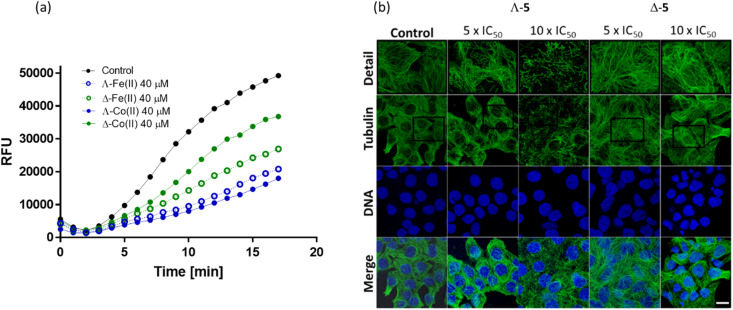
Effects on tubulin; (a) fluorescence growth (Ex/Em = 360/420 nm) reflecting tubulin polymerization was recorded in the absence (black) and in the presence of growing concentrations of Λ-5 (blue) and Δ-5 (green) at 37 °C. Comparative data for the isostructural Fe(ii) compound^16^ is included; (b) confocal fluorescence images. HCT116 cells were treated with Co(ii) triplexes at the given concentrations for 6 h. Microtubules were immunostained with primary anti-α-tubulin antibody and Alexa Fluor® 488 conjugated secondary antibody. Cell nuclei were counterstained with DAPI. Top panel shows detailed zoomed-in sections of each tubulin channel. Scale bar represents 20 μm.

Inhibition of tubulin polymerization is readily measured *in vitro via* a commercial fluorescence assay [Fig. 7(a)]. The Co(ii) compound Λ-5 inhibited tubulin polymerization far more efficiently (only 36% polymerization *vs.* control at 40 μM) than the Δ enantiomer (75% polymerization). This enantiomeric difference is substantially more pronounced than that observed for the Fe(ii) analogues (Fig. 7(a)) where the two enantiomers have similar performance in this assay.^16^

Visualization of the effects on cellular microtubule networks within cells was achieved *via* confocal fluorescence imaging. Cells were exposed to relatively high concentrations (5× and 10 × IC_50_ values) of enantiomers 5 for a relatively short period (6 h) before fixing and immunostaining (Fig. 7(b)).

Consistent with the *in vitro* data [Fig. 7(b)], Λ-5 induced substantial changes in the tubulin network of the HCT116 cells, at 5 × IC_50_ (3.5 μM); tangled microtubules are evident within the highlighted detailed sections (top row), and the impact increased at the higher concentration. In contrast, typical microtubule networks are visible in cells exposed to Δ-5 at 5 × IC_50_ (17.5 μM), while even at 35 μM the disturbance is subtle.

Actin filaments also play significant roles in cytoskeletal structures, but here the effects of both enantiomers of 5 were very modest, as evidenced by similar assays and confocal microscopy studies (Fig. S22 and S23†); actin filaments showed no marked changes upon cell exposure to 10 × IC_50_ concentrations of either compound.

## Conclusions

Using a range of self-assembly, oxidation and ion exchange processes, we synthesized the first examples of optically pure Co(ii) and Co(iii) metallohelices, including water-soluble and stable enantiomer pairs 5 and 6. The bidirectional ligand strand arrangement leads to distinctly amphipathic architectures. Notably, both metal oxidation states – rather than just Co(iii) – give inert (water-stable) species. An assessment of the redox chemistry of these compounds and their performance in cancer cells of various kinds indicate that the Co(iii) is reduced to Co(ii) *e.g. in cellulo* and in media.

There are similarities in the responses of HCT116 cells to treatment with the Co(ii) compound Λ-5 and the previously reported Fe(ii) compound Λ-Fe^II^; both induce G2-M arrest in the cell cycle, are more potent antiproliferative agents than their Δ-configured counterparts, and disrupt microtubule architectures within the cells. However, Λ-5 is *ca.* five times more potent than Λ-Fe^II^, and this difference does not appear to correlate with cellular uptake. In contrast, the enantiomer Δ-5 induces G1 arrest, similarly but more effectively than Δ-Fe^II^, again corresponding with the ten-times lower IC_50_ for the cobalt compound, which does not correlate with altered uptake kinetics.

In contrast to the previously reported iron system Δ-Fe^II^, and consistent with the cell cycle data, Δ-5 induces H2AX phosphorylation in HCT116 cells, indicating a DNA damage mechanism. Correspondingly, Δ-5 inhibits the topoisomerase I-catalysed relaxation of supercoiled plasmid DNA. The differences in the charge distributions of the Co(ii) metallohelices with respect to the Fe(ii) systems may lead to different binding interactions, but it seems very likely that redox chemistry plays a role. Given the greatly differing effects *in cellulo* of the two enantiomers of 5, it is highly plausible that Δ-5 catalyses oxidative damage *via* non-covalent interactions at specific binding sites in DNA, akin to the action of naturally-occurring substrate-specific metalloenzymes.^58^ In this context we note the very recent report of a new Cu(ii) peptide helicate that binds with high selectivity to DNA three-way junctions and selectively cleaves DNA at replication sites.^59^ The metal-based superoxide Cu–O_2_˙^−^ mechanism proposed therein is not available here since Δ-5 is inert, and the coordination sphere is saturated. Instead, we propose that binding of Δ-5 to negatively charged DNA would facilitate oxidation to Co(iii) *e.g.* by ROS, in turn leading to outer-sphere oxidative DNA damage and release of the Co(ii) metallohelix.

As indicated by cell cycle and TCRP profiles, Λ-5 and Λ-6 operate by a different mechanism to the Δ compounds, inhibiting tubulin polymerization and causing significant changes to the microtubule architecture of the cancer cells.

Overall, we conclude that the new cobalt metallohelices have enantiomer-dependent mechanisms of action, with the Δ compounds causing DNA damage while Λ acts on tubulin architecture. Both mechanisms are worthy of further study, and at this point we also note that derivatives of the enantiomerically pure compounds 5 should be readily prepared by self-assembly, providing routes to optimization and probing of structure–activity relationships.

## Data availability

All experimental details and characterisation data can be found in the ESI.†

## Author contributions

H. S., V. B., N. J. R., and P. S. designed research; H. S., N. J. R., H. K., J. C., J. M., T. R., M. P., S. S. and X. Z. performed research; H. S., J. M., R. P., V. B., N. J. R., and P. S. analysed data; and H. S., V. B., N. J. R., and P. S. wrote the paper.

## Conflicts of interest

There are no conflicts to declare.

## Supplementary Material

SC-015-D4SC02541E-s001
