# Supplementary material for: Dicobalt(ii) helices kill colon cancer cells via enantiomer-specific mechanisms; DNA damage or microtubule disruption
Source: Chem Sci. 2024 Jun 14;15(28):11029–37. doi: 10.1039/d4sc02541e (PMC11253168; doi:10.1039/d4sc02541e)
Supplement: SC-015-D4SC02541E-s001 [file SC-015-D4SC02541E-s001.pdf]

Electronic Supplementary Material (ESI) for Chemical Science.
This journal is © The Royal Society of Chemistry 2024

Electronic Supplementary Information

Dicobalt(II) helices kill colon cancer cells via enantiomer-specific mechanisms; DNA damage or microtubule disruption

Hualong Song*[a,b], Hana Kostrhunova[c], Jakub Cervinka[c,d], Julie Macpherson[b], Jaroslav Malina[c], Teena Rajan[b], Roger Phillips[e], Miles Postings[b], Samantha Shepherd[e], Xuejian Zhang[f], Viktor Brabec*[c,g], Nicola J. Rogers*[h], Peter Scott*[b]

a. Beijing Area Major Laboratory of Peptide and Small Molecular Drugs, Engineering Research Centre of Endogenous Prophylactic of Ministry of Education of China, School of Pharmaceutical Sciences, Capital Medical University, Beijing, 100069, China
b. Department of Chemistry, University of Warwick, Coventry, CV4 7AL, UK
c. Czech Academy of Sciences, Institute of Biophysics, Brno, Czech Republic
d. Faculty of Science, Department of Biochemistry, Masaryk University, Brno, Czech Republic
e. Department of Pharmacy, University of Huddersfield, Huddersfield HD1 3DH, U.K.
f. College of Chemistry, State Key Laboratory of Elemento-Organic Chemistry, Nankai University, Tianjin, 300071, China
g. Department of Biophysics, Palacky University, Olomouc, Czech Republic
h. Department of Chemistry, Hong Kong Baptist University, Kowloon Tong, Hong Kong SAR, China

*Hualong Song; Viktor Brabec; Nicola J. Rogers; Peter Scott

Email: hualong.song@ccmu.edu.cn; brabec@ibp.cz; nicolarogers@hkbu.edu.hk; peter.scott@warwick.ac.uk

Contents

1. Synthesis 3
2. NMR Spectra 9
3. High resolution ESI mass spectra 12
4. Stability tests in aqueous media 15
5. DFT Calculations 17
6. Redox properties of the cobalt triplex metallohelices 25
7. Cancer cell studies 27
8. References 36

1. Synthesis

All solvents and chemicals purchased from commercial sources (Sigma-Aldrich, Acros, Fisher Scientific or Alfa Aesar) were used without further purification unless otherwise stated. Sodium hydride dispersions in mineral oil were placed in a Schlenk vessel under an inert atmosphere and washed three times with diethyl ether to remove the oil, then dried and stored under argon in an MBraun dry box. Where appropriate, reactions were carried out under argon using a dual manifold argon/vacuum line and standard Schlenk techniques or in an MBraun dry box. Necessary solvents were dried by heating to reflux for 3 d under dinitrogen over the appropriate drying agents (potassium for tetrahydrofuran and sodium/potassium alloy for diethyl ether) and degassed before use. Tetrahydrofuran and diethyl ether were additionally pre-dried over sodium wire. Dried solvents were stored in glass ampoules under argon. All glassware and cannulae were stored in an oven at > 375 K.

Deuterated solvents were purchased from Sigma-Aldrich and Cambridge Isotope Laboratories. NMR spectra were recorded on Bruker Spectrospin 300/400/500 MHz spectrometers. Routine NMR assignments were confirmed by ^{1}H-^{1}H (COSY) and ^{1}H-^{13}C (HSQC) correlation experiments where necessary. The spectra were internally referenced using the residual protio solvent ($CDCl_3$, CD_3CN etc.) resonance relative to tetramethylsilane ($\delta = 0$ ppm). ESI mass spectra were recorded on an Agilent Technologies 1260 Infinity spectrometer or a Bruker Daltonics MicroTOF spectrometer. Infra-Red spectra were measured using a Bruker Alpha-P FTIR spectrometer. Elemental analyses were performed by Medac Ltd. Chobham, Surrey GU24, 8JB, UK.

The ligand precursors (*S/R*)-2-(2,2'-bipyridin-5-ylmethoxy)-1-phenylethanamine were prepared by the reported literatures.[1]

S$_{C}$,Λ_{Co},HHT-[Co_2L_3][ClO_4]$_4$ (Λ-3)

Cobalt(II) perchlorate hexahydrate (54.9 mg, 0.15 mmol) was added to a stirred solution of the 2-pyridinecarboxaldehyde (24.0 mg, 0.23 mmol) and (*S*)-2-(2,2'-bipyridin-5-ylmethoxy)-1-phenylethanamine (68.6 mg, 0.23 mmol) in acetonitrile (15 ml) at ambient temperature to give a bright yellow solution that was then heated to reflux for 48 h. The reaction mixture was

cooling down to ambient temperature and the solvent was removed *in vacuo* to give the desired bright yellow crystallite.

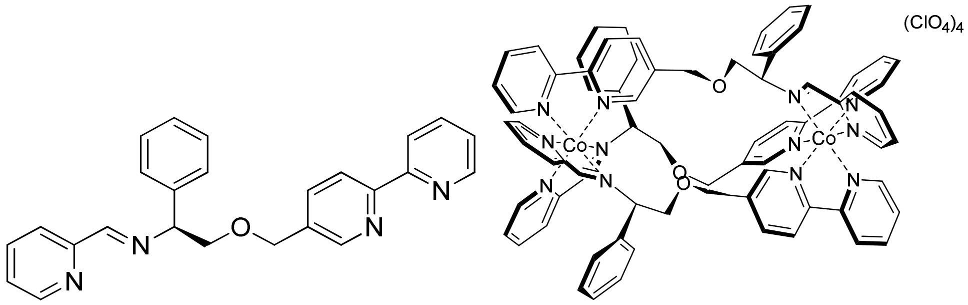

Yield 124 mg, 84 %

Elemental Analysis found (Calculated for $C_{75}H_{66}Cl_4Co_2N_{12}O_{19}\cdot 2H_2O$) % C 51.94 (51.92), H 3.94 (4.07), N 9.48 (9.69).

HRMS Calculated for $[L+H]^+$ *m/z* 395.1866, found *m/z* 395.1862; $[Co_2L_3]^{4+}$ *m/z* 325.1006, found *m/z* 325.0993; $[Co_2L_3][ClO_4]_2^{2+}$ *m/z* 750.1504, found *m/z* 750.1491;

IR *v* cm^{-1} 3541 (w), 3058 (w), 1638 (w), 1598 (m), 1472 (w), 1440 (w), 1307 (w), 1242 (w), 1068 (s), 930 (m), 861 (w), 841 (w), 750 (m), 697 (m) 619 (s).

R_c,Δ_{Co},HHT-$[Co_2L_3][ClO_4]_4$ (Δ-3)

Data as for *S*-enantiomer

Yield 114 mg, 77 %

Elemental Analysis found (Calculated for $C_{75}H_{66}Cl_4Co_2N_{12}O_{19}\cdot 4H_2O$) % C 50.97 (50.86), H 3.95 (4.21), N 9.25 (9.49).

S_{C},Λ_{Co},HHT-[Co_2L_3]Cl_4 (Λ-5)

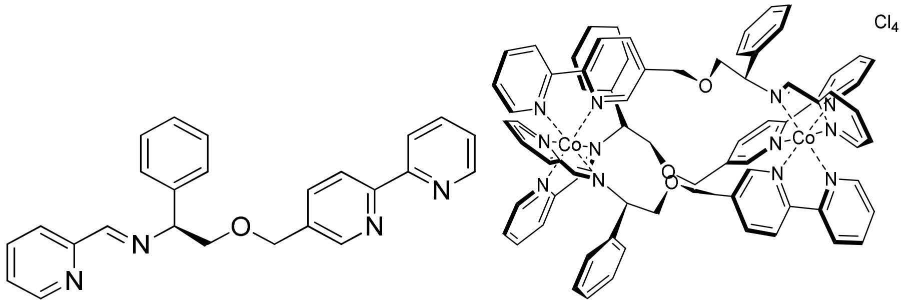

Cobalt(II) chloride (16.0 mg, 0.12 mmol) was added to a stirred solution of the 2-pyridinecarboxaldehyde (19.8 mg, 0.18 mmol) and (*S*)-2-(2,2'-bipyridin-5-ylmethoxy)-1-phenylethanamine (56.4 mg, 0.18 mmol) in methanol (20 ml) at ambient temperature to give a bright yellow solution that was then heated to reflux for 48 h. The solvent was removed *in vacuo* to give bright yellow crystals.

Yield 84 mg, 86 %

Elemental Analysis found (Calculated for $C_{75}H_{66}Cl_4Co_2N_{12}O_3 \cdot 8H_2O$) % C 56.84 (56.75), H 4.70 (5.21), N 10.42 (10.59).

HRMS Calculated for $[L+H]^+$ *m/z* 395.1866, found *m/z* 395.1861; $[Co_2L_3]^{4+}$ *m/z* 325.1006, found *m/z* 325.0991; $[Co_2L_3]Cl^{3+}$ *m/z* 445.1239, found *m/z* 445.1229;

IR *v* cm^{-1} 3310 (s), 3065 (s), 1637 (m), 1596 (s), 1567 (m), 1471 (s), 1438 (s), 1355 (w), 1310 (w), 1232 (w), 1169 (w), 1102 (s), 1076 (s), 1048 (s), 1014 (s), 932 (w), 843 (w), 790 (w), 753 (s), 698 (s).

R_{C},Δ_{Co},HHT-[Co_2L_3]Cl_4 (Δ-5)

Data as for *S*-enantiomer

Yield 86 mg, 88 %

Elemental Analysis found (Calculated for $C_{75}H_{66}Cl_4Co_2N_{12}O_3 \cdot 8H_2O$) % C 56.93 (56.75), H 4.57 (5.21), N 10.07 (10.59).

S_{C},Λ_{Co},HHT-[Co_2L_3][PF_6]$_6$ (Λ-4)

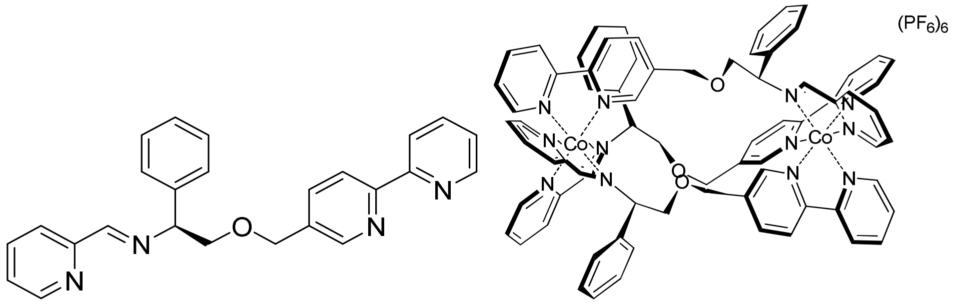

S_{C},Λ_{Co},HHT-[Co_2L_3][ClO_4]$_4$ (118.5 mg, 0.067 mmol) was dissolved in 15 ml CH_3CN, followed by dropwise addition of ammonium cerium(IV) nitrate (54.8 mg, 0.1 mmol) in CH_3CN (5 ml). A precipitate was observed immediately, and the suspension was stirred for 1 h, then filtered, washed with CH_3CN and dissolved in water (15 ml). The addition of NH_4PF_6 (0.598 g, 3.67 mmol) into the solution led to the formation of an orange precipitate which was filtered, washed with H_2O and dissolved in CH_3CN before the solvent was removed under vacuum to give the product as an orange crystallite.

Yield 143 mg, 89 %

^{1}H NMR (500 MHz, 298 K, CD_3CN) δ_H ppm 9.42 (s, 1H, HC=N), 9.35 (s, 1H, HC=N), 9.30 (s, 1H, bpy), 9.26 (s, 1H, bpy), 8.99 (s, 1H, HC=N), 8.80 (dd, $^{3}J_{HH}$ = 7.5 Hz, 1H, bpy), 8.69 (m, 2H, bpy), 8.65 (d, $^{3}J_{HH}$ = 7.8 Hz, 1H, bpy), 8.60 (t, $^{3}J_{HH}$ = 7.7 Hz, 1H, py), 8.49 (m, 2H, py), 8.44 (t, $^{3}J_{HH}$ = 7.8 Hz, 1H, py), 8.32 (t, $^{3}J_{HH}$ = 11.3 Hz, 2H, py), 8.21 (t, $^{3}J_{HH}$ = 7.6 Hz, 1H, py), 8.18 (t, $^{3}J_{HH}$ = 7.8 Hz, 1H, py), 8.11 (m, 2H, py/bpy), 7.99 (d, $^{3}J_{HH}$ = 8.2 Hz, 1H, bpy), 7.87 (t, $^{3}J_{HH}$ = 6.6 Hz, 1H, bpy), 7.84–7.60 (m, 8H, bpy/py), 7.58 (s, 1H, bpy), 7.31 (t, $^{3}J_{HH}$ = 7.3 Hz, 1H, Ph), 7.24–7.15 (m, 3H, Ph/bpy), 7.14–7.03 (m, 4H, Ph/bpy), 6.96 (d, $^{3}J_{HH}$ = 5.9 Hz, 1H, bpy), 6.86 (m, 3H, Ph/bpy), 6.74 (m, 3H, Ph/bpy), 6.45 (brs, 6H, Ph), 5.53–5.35 (m, 3H, OCH_2-bpy/CHPh), 5.30 (d, $^{2}J_{HH}$ = 13.2 Hz, 1H, OCH_2-bpy), 4.64 (m, 2H, OCH_2-bpy), 4.52 (d, $^{2}J_{HH}$ = 13.0 Hz, 1H, OCH_2-bpy), 4.45 (t, $^{3}J_{HH}$ = 11.6 Hz, 1H, CH_2-CHPh), 4.32 (m, 3H, CH_2-CHPh/CHPh), 4.22 (dd, J_{HH} = 11.1, 3.1 Hz, 1H, CHPh), 3.78 (dd, J_{HH} = 11.0, 4.1 Hz, 1H, CH_2-CHPh), 3.52 (dd, J_{HH} = 12.1, 3.4 Hz, 1H, CH_2-CHPh), 3.42 (d, $^{3}J_{HH}$ = 9.6 Hz, 1H, CH_2-CHPh).

^{13}C {^{1}H}q NMR (125 MHz, 298 K, CD_3CN) δ_C ppm 178.6, 178.5, 177.3 (HC=N), 157.3, 157.1, 156.0, 155.7, 155.1 (q, bpy), 155.0,154.9, 154.9 (q, py), 154.5, 153.9, 153.3, 152.8, 152.7,

152.3, 151.9, 151.6, 151.4 (bpy), 146.2, 145.8, 145.6, 145.0, 144.7, 144.5, 144.5, 144.4, 144.2 (py), 141.8, 141.1 (q, bpy), 136.4, 136.0, 134.4, 134.3, 134.0, 133.7 (bpy), 132.4 (q, Ph), 132.2, 132.1, 131.9 (py), 131.0, 130.9 (Ph), 130.7 (q, Ph), 130.6, 130.5 (Ph), 130.4 (q, Ph), 130.1, 130.0 (Ph), 127.9, 127.7, 127.2, 127.1, 126.4, 126.0 (bpy), 73.6, 73.1, 70.6 ($\underline{C}$HPh), 69.9, 69.9, 69.5 ($\underline{C}H_2$-bpy), 69.1, 69.0, 68.8 ($\underline{C}H_2$-CHPh).

Elemental Analysis found (Calculated for $C_{75}H_{66}Co_2F_{36}N_{13}O_3P_6 \cdot 12H_2O$) % C 37.17 (37.51), H 3.32 (3.78), N 7.90 (7.58).

HRMS Calculated for $[Co_2L_3][PF_6]_3^{3+}$ *m/z* 578.4318, found *m/z* 578.4335

IR ν cm^{-1} 3322 (w), 3050 (w), 1599 (w), 1472 (w), 1239 (w), 1172 (s), 987 (s), 831 (m), 720 (s), 695 (s), 546 (m), 437 (s).

R_C,Δ_{Co},HHT-$[Co_2L_3][PF_6]_6$ (Δ-4)

Data as for *S*-enantiomer

Yield 132 mg, 82 %.

Elemental Analysis found (Calculated for $C_{75}H_{66}Co_2F_{36}N_{13}O_3P_6 \cdot 4H_2O$) % C 39.77 (39.91), H 3.47 (3.30), N 7.64 (8.07).

S_C,Λ_{Co},HHT-$[Co_2L_3]Cl_6$ (Λ-6)

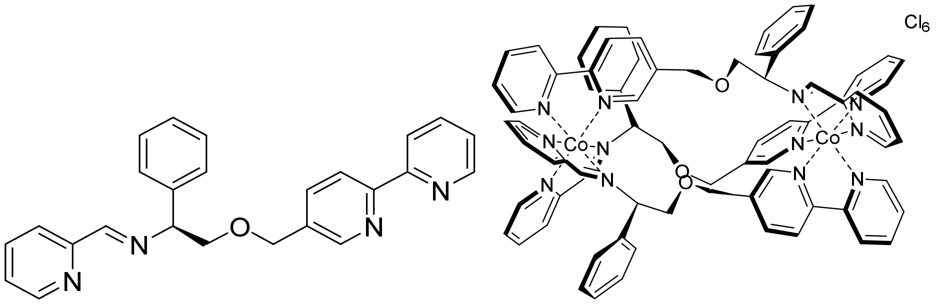

S_C,Λ_{Co},HHT-$[Co_2L_3][PF_6]_6$ (132.4 mg, 0.055 mmol) and Amberlite® IRA-400 (chloride form) resin (1 g) were added into 20 ml H_2O. The reaction mixture was stirred overnight. The

suspension was filtered to remove the resin and the solvent was removed under vacuum to give the product as a pale yellow solid.

Yield 91 mg, 92 %

^{1}H NMR (500 MHz, 298 K, D_2O) δ_H ppm 9.42 (s, 1H, bpy), 9.39 (s, 1H, bpy), 8.80 (d, $^{3}J_{HH}$ = 7.6 Hz, 1H, bpy), 8.73 (d, $^{3}J_{HH}$ = 8.2 Hz, 1H, bpy), 8.70 (d, $^{3}J_{HH}$ = 8.1 Hz, 1H, bpy), 8.67 (d, $^{3}J_{HH}$ = 7.6 Hz, 1H, bpy), 8.59 (t, $^{3}J_{HH}$ = 7.8 Hz, 1H, py), 8.55 – 8.45 (m, 2H, py), 8.41 (t, $^{3}J_{HH}$ = 7.9 Hz, 1H, py), 8.38 – 8.31 (m, 2H, py), 8.29-8.17 (m, 3H, py), 8.14 (m, 2H, py/bpy), 7.96 (d, $^{3}J_{HH}$ = 7.9 Hz, 1H, bpy), 7.89 (d, $^{3}J_{HH}$ = 8.0 Hz, 1H, bpy), 7.87-7.80 (m, 2H, py), 7.78 – 7.69 (m, 3H, bpy/py), 7.63 (t, $^{3}J_{HH}$ = 6.8 Hz, 2H, bpy), 7.51 (s, 1H, bpy), 7.33-7.26 (m, 3H, bpy/Ph), 7.22 (t, $^{3}J_{HH}$ = 6.3 Hz, 2H, bpy), 7.17 (t, $^{3}J_{HH}$ = 7.5 Hz, 2H, Ph), 7.12 – 7.05 (m, 2H, bpy/Ph), 6.97 (t, $^{3}J_{HH}$ = 6.4 Hz, 2H, bpy), 6.83 (t, $^{3}J_{HH}$ = 7.6 Hz, 2H, Ph), 6.73 (t, $^{3}J_{HH}$ = 7.7 Hz, 2H, Ph), 5.53 (dd, *J* = 11.1, 4.1 Hz, 1H, C<u>H</u>Ph), 5.45 (d, $^{2}J_{HH}$ = 13.3 Hz, 2H, OC<u>H</u>$_2$-bpy), 5.34 (d, $^{2}J_{HH}$ = 13.2 Hz, 1H, OC<u>H</u>$_2$-bpy), 4.64 – 4.48 (m, 5H, OC<u>H</u>$_2$-bpy/C<u>H</u>$_2$-CHPh overlapping with D_2O), 4.43 (t, $^{3}J_{HH}$ = 11.1 Hz, 1H, C<u>H</u>$_2$-CHPh), 4.36 (dd, *J* = 10.6, 2.8 Hz, 1H, C<u>H</u>Ph), 4.29 (dd, *J* = 11.0, 3.2 Hz, 1H, C<u>H</u>Ph), 3.78 (dd, *J* = 11.1, 4.0 Hz, 1H, C<u>H</u>$_2$-CHPh), 3.49 (dd, *J* = 12.1, 3.2 Hz, 1H, C<u>H</u>$_2$-CHPh), 3.39 (dd, *J* = 12.3, 2.9 Hz, 1H, C<u>H</u>$_2$-CHPh).

$^{13}C\{^{1}H\}$ NMR (125 MHz, 298 K, D_2O) δ_C ppm 157.2, 156.9, 156.0, 155.3, 154.8, 154.8 (q, bpy), 154.6, 154.5, 154.4 (q, py), 153.7, 153.2, 152.4, 152.3, 151.7, 151.4, 151.2, 150.8, 150.4 (bpy), 145.3, 145.2, 145.1, 145.0, 144.6, 144.5, 144.4, 144.3, 144.2 (py), 141.0, 140.6, 140.5 (q, bpy), 135.1, 134.7, 133.6, 133.5, 133.2, 133.1 (bpy), 131.7 (q, Ph), 131.6, 131.5, 131.4 (py), 130.8, 130.7, 130.4, 130.2, 129.9, 129.8, 129.7 (Ph), 129.6 (q, Ph), 127.5, 127.2, 126.9, 126.1, 125.6 (bpy), 73.3, 72.9, 70.1 (<u>C</u>HPh), 69.5, 69.4, 68.8 (<u>C</u>H$_2$-bpy), 68.6, 68.3, 68.1(<u>C</u>H$_2$-CHPh).

Elemental Analysis found (Calculated for $C_{75}H_{66}Cl_6Co_2N_{12}O_3\cdot 16H_2O$) % C 49.53 (49.98), H 4.96 (5.48), N 9.36 (9.33).

HRMS Calculated for $[Co_2L_3\text{-}2H]Cl_2^{2+}$ *m/z* 685.1629, found *m/z* 685.1634; $[Co_2L_3\text{-}2H]Cl^{3+}$ *m/z* 444.4520, found *m/z* 444.4529; $[Co_2L_3\text{-}3H]^{3+}$ *m/z* 432.4598, found *m/z* 432.4585;

IR *v* cm^{-1} 3332 (s), 3031 (s), 1628 (w), 1597 (m), 1470 (w), 1444 (s), 1408 (w), 1377 (w), 1321 (w), 1240 (m), 1175 (w), 1116 (w), 1066 (s), 1029 (m), 1009 (m), 937 (w), 844 (w), 754 (m), 699 (s).

R_c,Δ_{Co},HHT-[Co_2L_3]Cl_6 (Δ-6)

Data as for *S*-enantiomer

Yield 88 mg, 89 %

Elemental Analysis found (Calculated for $C_{75}H_{66}Cl_6Co_2N_{12}O_3 \cdot 14H_2O$) % C 51.23 (51.00), H 5.14 (5.36), N 9.74 (9.52).

2. NMR Spectra

Typical of high spin Co(II) there are large hyperfine shifts and moderate broadening, with short electronic relaxation times (ps) and large magnetic anisotropy. Several broader resonances [*ca* 200 Hz (FWHM), corresponding to T_2^* = 1.6 ms at 7 T] are assigned to the bipyridyl, pyridyl, and benzylic protons most proximate to the metal centers for the three chemically inequivalent ligand strands.

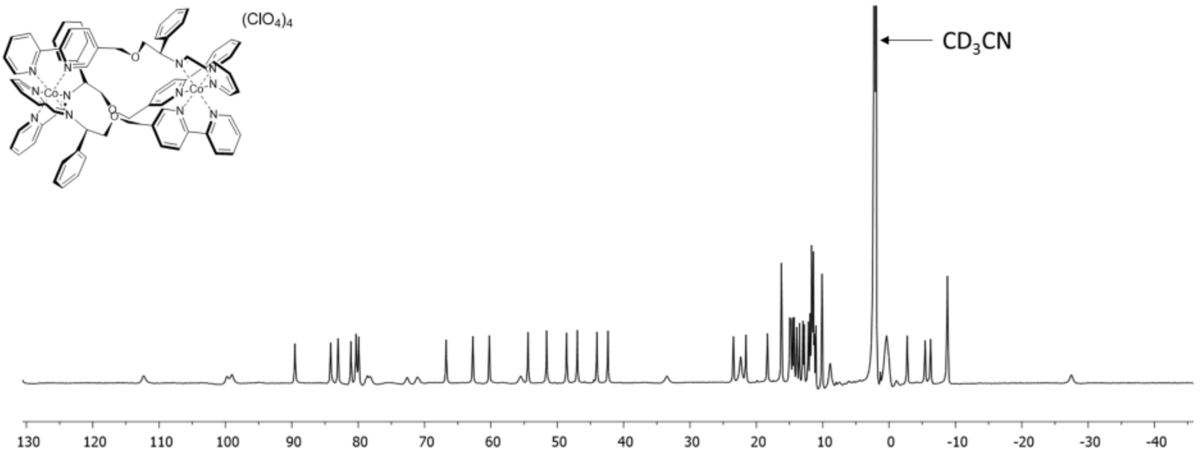

Figure S1. ^{1}H (300 MHz, CD_3CN, 298K) NMR spectrum of (S_c,Λ_{Co})-HHT-[Co_2**L**$_3$][ClO_4]$_4$ (**Λ-3**)

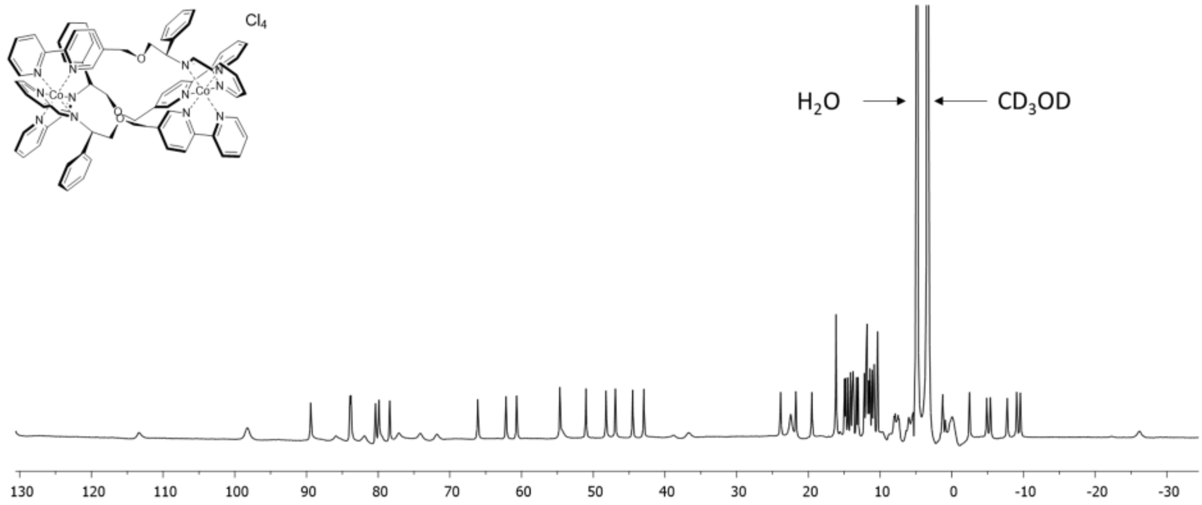

Figure S2. ^{1}H (300 MHz, CD_3OD, 298K) NMR spectrum of (S_c,Λ_{Co})-HHT-[Co_2**L**$_3$]Cl_4 (**Λ-5**)

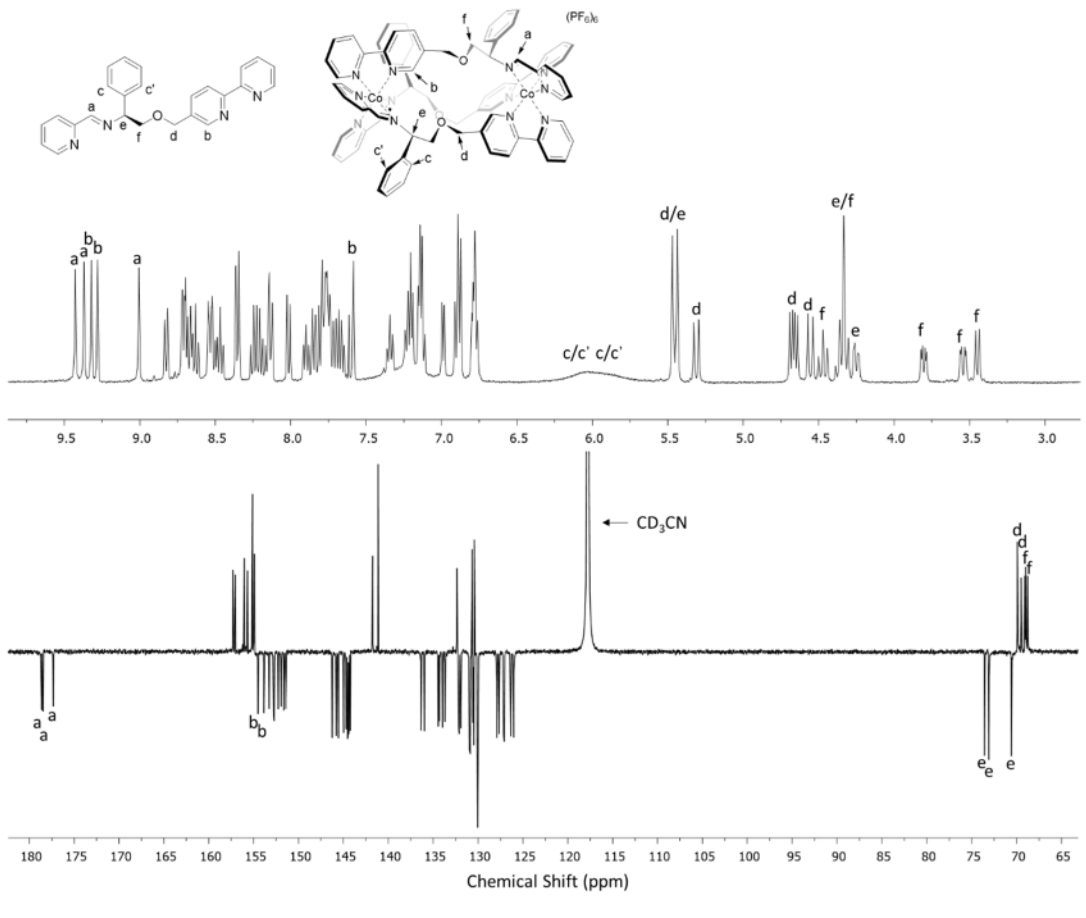

Figure S3. ^{1}H (500 MHz, CD_3CN, 298K) and ^{13}C (125 MHz, CD_3CN, 298K) NMR spectra of S_c,Λ_{Co},HHT-[Co_2**L**$_3$][PF_6]$_6$ (**Λ-4**)

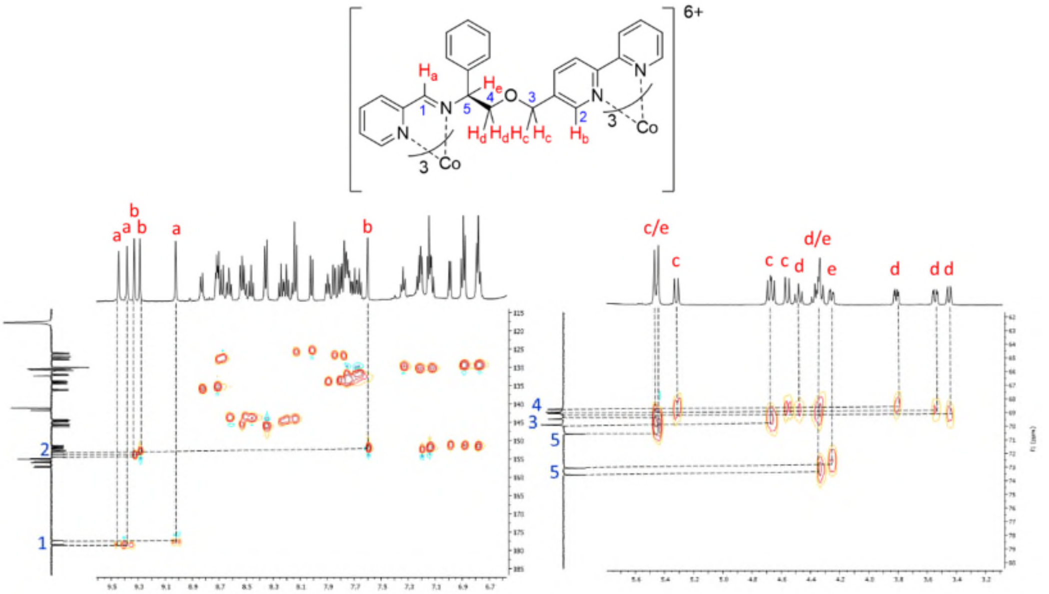

Figure S4. 2D ^{1}H-^{13}C HSQC (500 MHz/125 MHz, CD_3CN, 298K) NMR spectra of S_c,Λ_{Co},HHT-[Co_2**L**$_3$][PF_6]$_6$ (**Λ-4**)

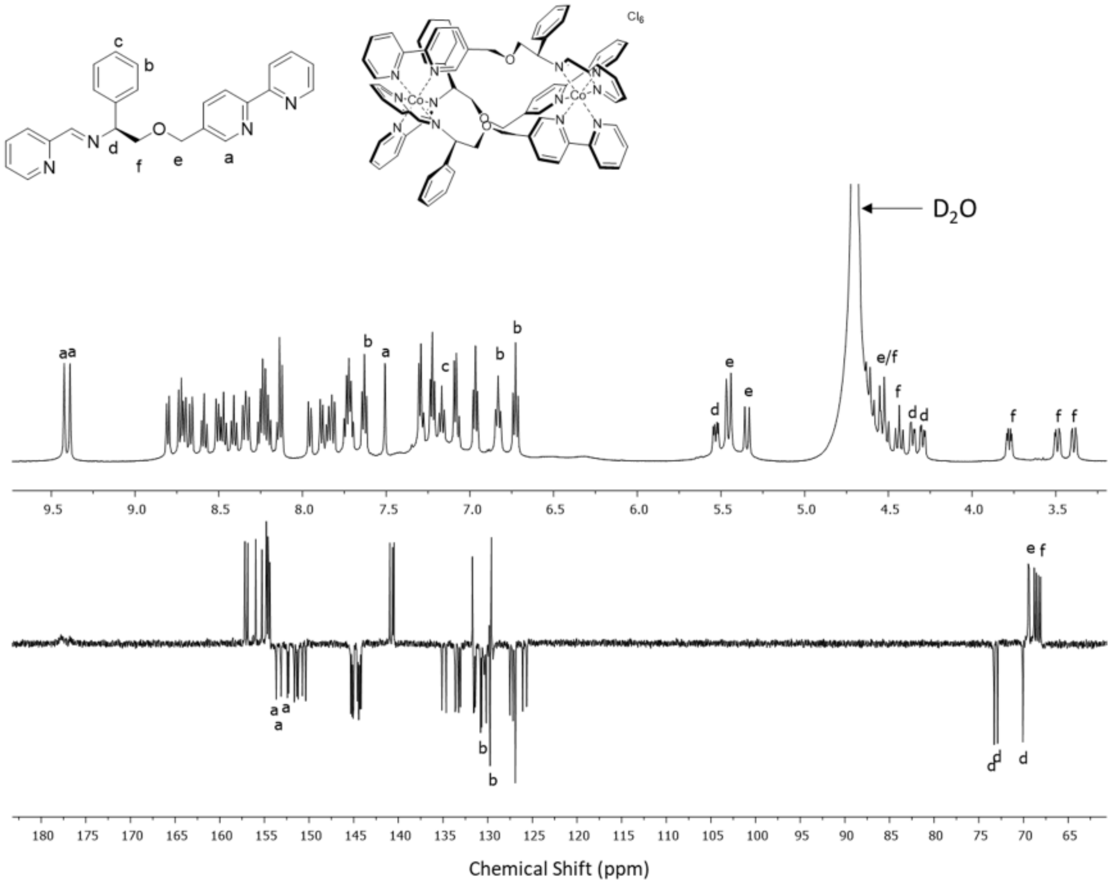

Figure S5. ^{1}H (500 MHz, D_2O, 298K) and ^{13}C (125 MHz, D_2O, 298K) NMR spectra of S_c,Λ_{Co},HHT-[Co_2**L**$_3$]Cl_6 (**Λ-6**)

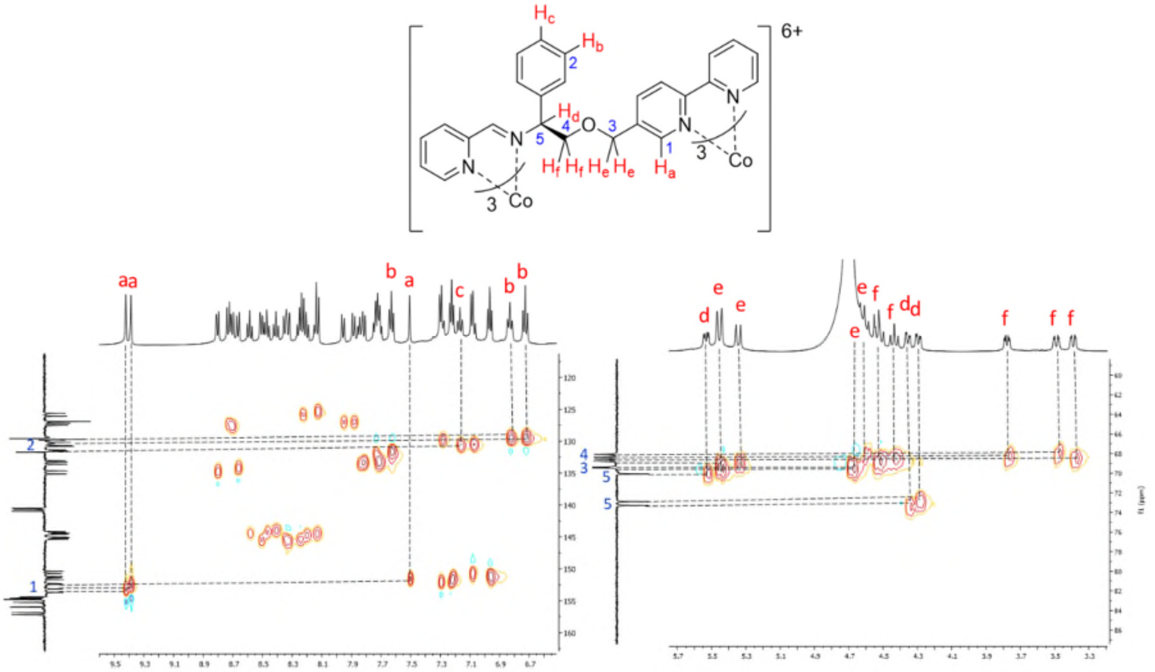

Figure S6. 2D ^{1}H-^{13}C HSQC (500 MHz/125 MHz, D_2O, 298K) NMR spectra of S_c,Λ_{Co},HHT-[Co_2**L**$_3$]Cl_6 (**Λ-6**)

3. High resolution ESI mass spectra

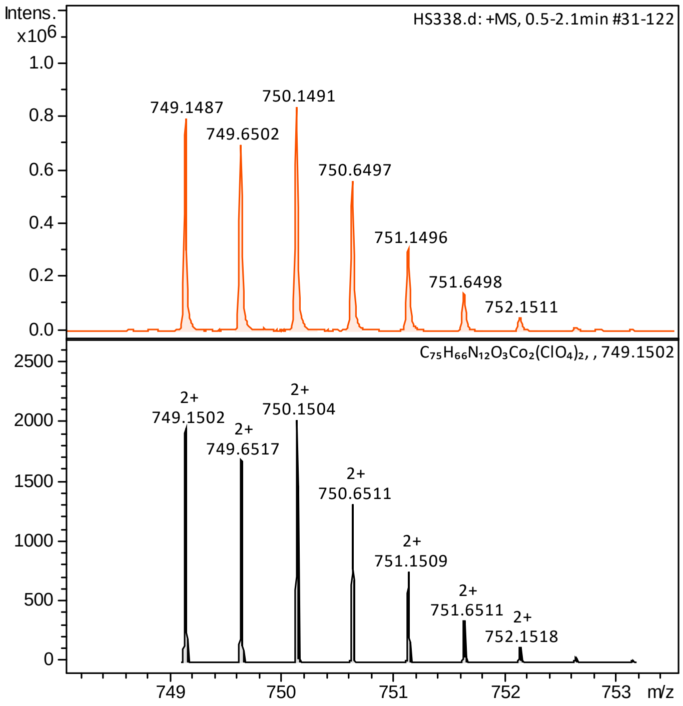

Figure S7. High resolution ESI mass spectrum of (S_c,Λ_{Co})-[Co_2**L**$_3$][[ClO_4]$_4$ (**Λ-3**) showing the observed z = +2 charge (top), compared to the theoretical isotope pattern (bottom).

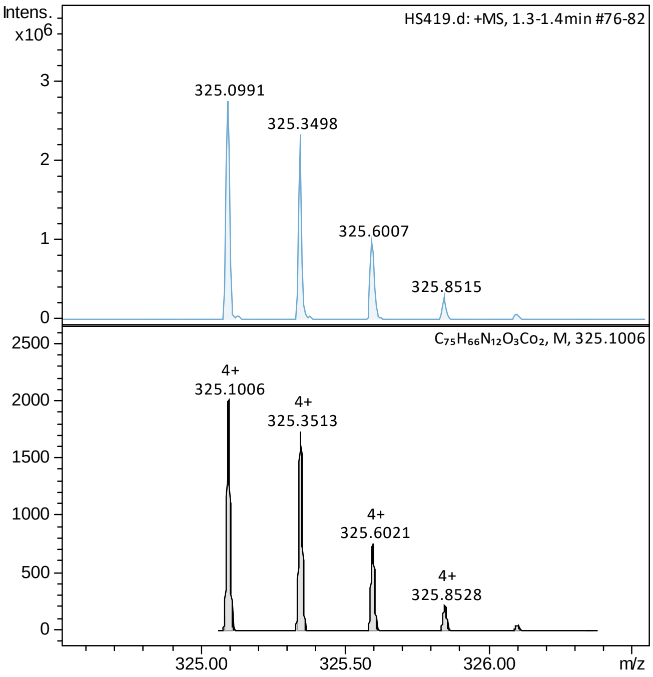

Figure S8. High resolution ESI mass spectrum of (S_c,Λ_{Co})-[Co_2**L**$_3$]Cl_4 (**Λ-5**) showing the observed z = +4 charge (top), compared to the theoretical isotope pattern (bottom).

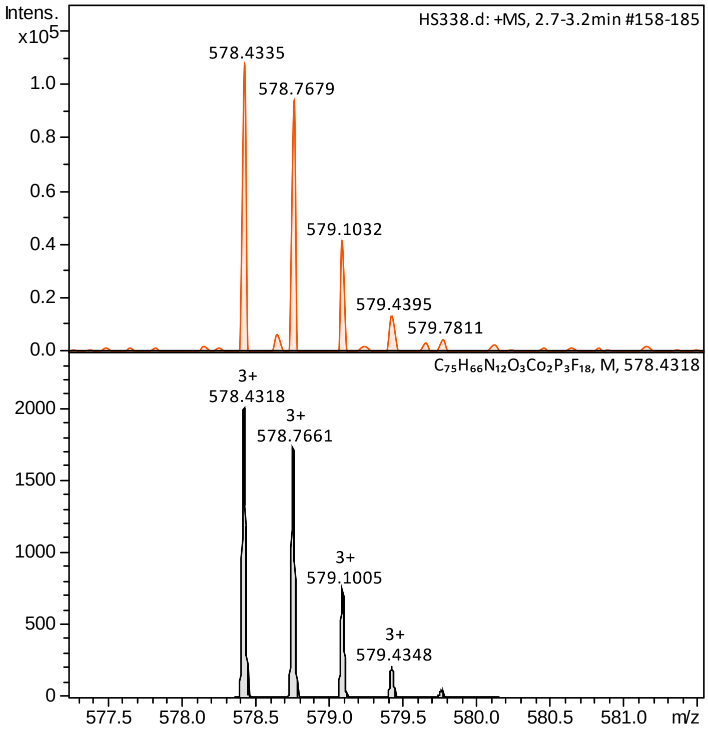

Figure S9. High resolution ESI mass spectrum of (S_c,Λ_{Co})-[Co_2**L**$_3$][[PF_6]$_6$ (**Λ-4**) showing the observed z = +3 charge (top), compared to the theoretical isotope pattern (bottom).

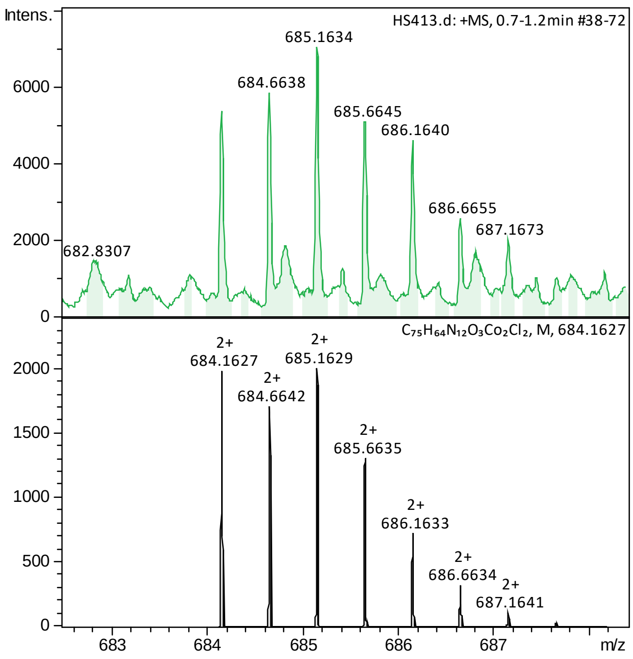

Figure S10. High resolution ESI mass spectrum of (S_c,Λ_{Co})-[Co_2**L**$_3$-2H]Cl_2^{2+} (**Λ-6**) showing the observed z = +2 charge (top), compared to the theoretical isotope pattern (bottom).

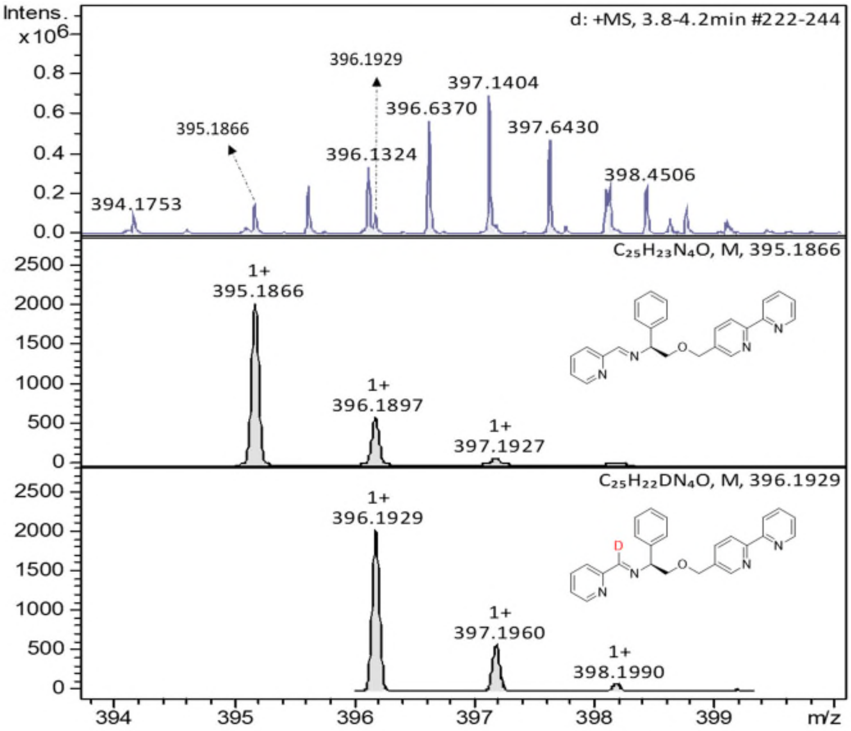

Figure S11. High resolution mass spectrum showed the imine deuterium exchange of (S_c,Λ_{Co})-[Co_2**L**$_3$]Cl_6 (**Λ-6**), the complex was dissolved in D_2O before the measurement, both ligand and deuterated ligand were observed in spectrum. [Ligand+H]$^+$ m/z 395.1866, 73.3% (peak intensity 148070); [Deuterated ligand+H]$^+$ m/z 396.1929, 26.7% (peak intensity 96641).

4. Stability tests in aqueous media

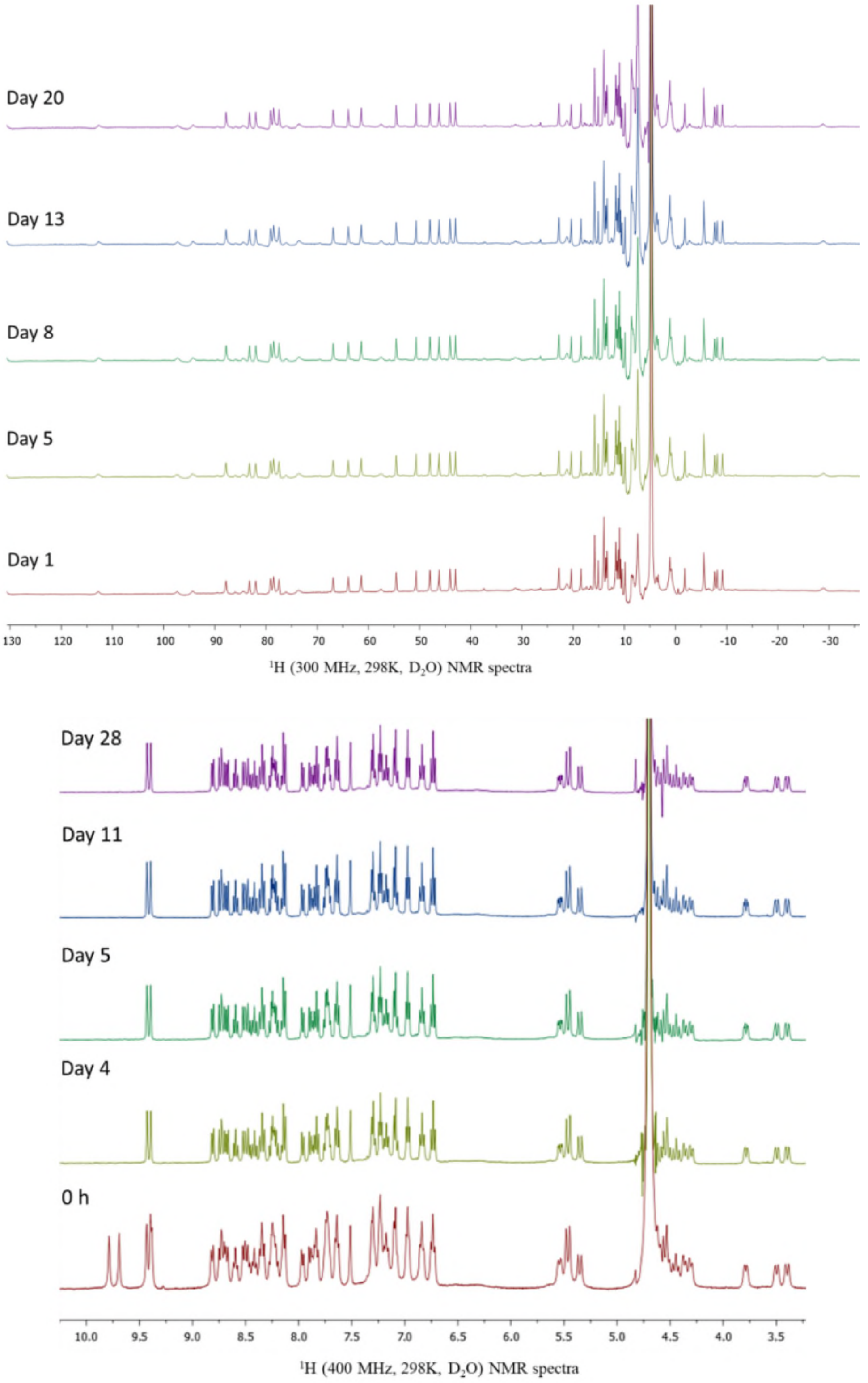

Figure S12. Stability of S_c,Λ_{Co},HHT-[Co_2**L**$_3$]Cl_4 (**Λ-5**) (upper) and S_c,Λ_{Co},HHT-[Co_2**L**$_3$]Cl_6 (**Λ-6**) (lower) in D_2O

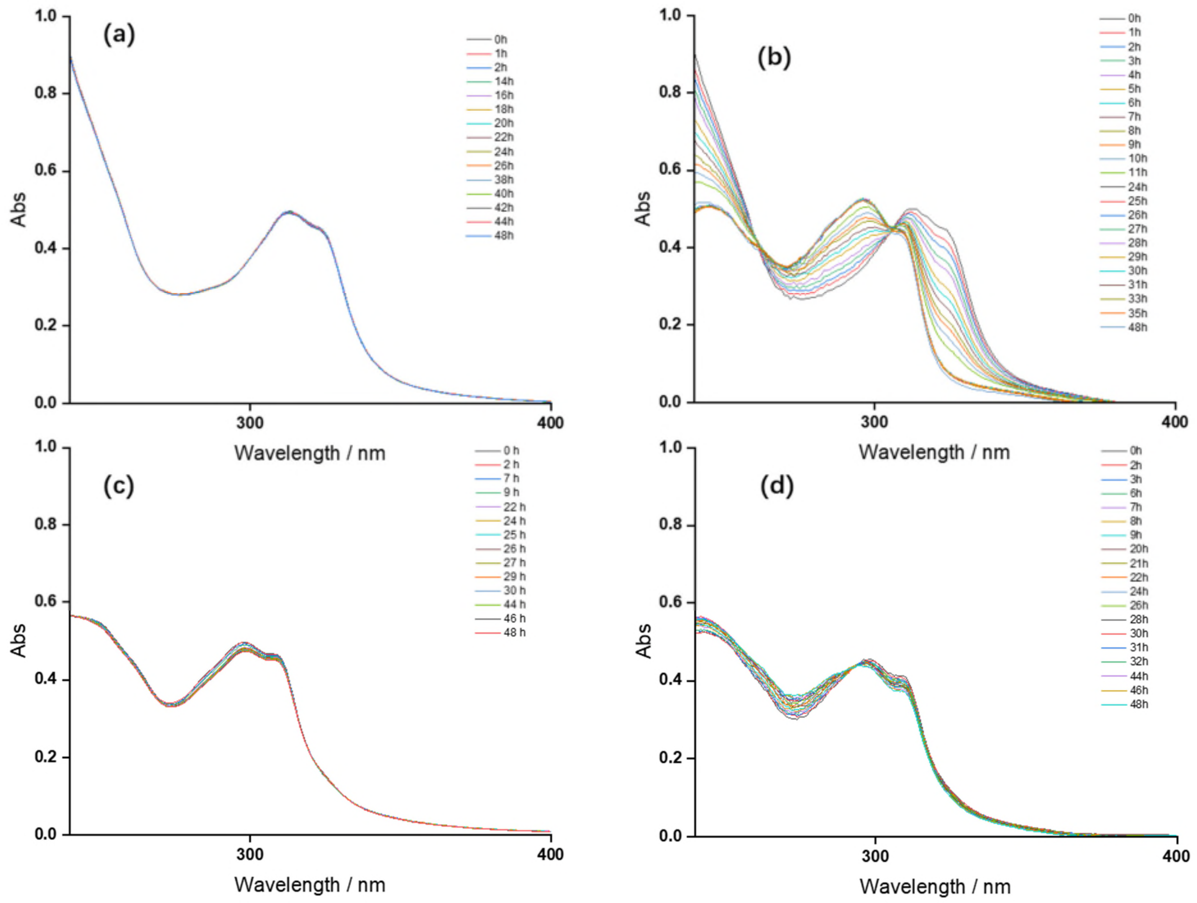

Figure S13. Stability studies by UV/vis at 37 °C: (a) Λ-Co(III) (10μM) in PBS; (b) Λ-Co(III) (10μM) in cell media (DMEM+10%FBS) showing isosbestic points for reduction of Co(III) to Co(II); (c) Λ-Co(II) (10μM) in PBS; (d) Λ-Co(II) (10μM) in cell media (DMEM+10%FBS).

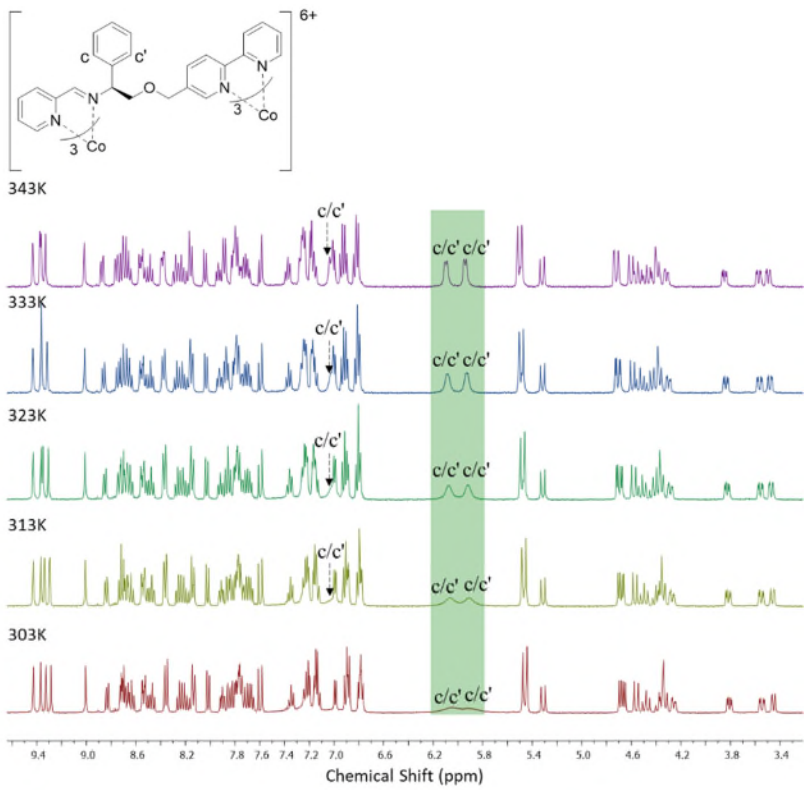

Figure S14. Variable temperature ^{1}H NMR spectra of (S_c,Λ_{Co})-[Co_2**L**$_3$][[PF_6]$_6$ (**Λ-4**) (400 MHz, CD3CN)

5. DFT Calculations

5.1 Geometry optimisation

All calculations were carried out using ORCA program.[2] Initial monometallic sub-structures for each metallohelices were created from existing crystallographic fragments and optimised without symmetrical restrictions. The ground state geometry optimisation were performed using density functional theory (DFT) with B3LYP functional, employing a triple-zeta def2-TVZP basis set for metal atoms and def2-SVP basis set for other atoms in the gas phase.[3] Dispersion was also addressed in the optimisation using Grimme's D3BJ dispersion correction.[4, 5] The local minima of optimised structures were confirmed by the vibrational frequency analysis.

The starting geometries of bimetallic metallohelices were assembled from the corresponding optimised monometallic fragments, and further optimised using the same basis set as before. Solvation energy correction was performed in water using SMD continuum solvation model. The (U)BP86-D3(BJ) functional with a mixed basis set of def2-TVZP for metal ions and def2-

SVP for other atoms was used for geometry optimisation in solution phase and single point energy calculations.

5.2 Electrostatic potential (ESP)

Quantitative calculation of electrostatic potential surfaces of metallohelices were obtained using Multiwfn program[6] using B3LYP/G-31G** level and corresponding plots were generated using the VMD program.[7]

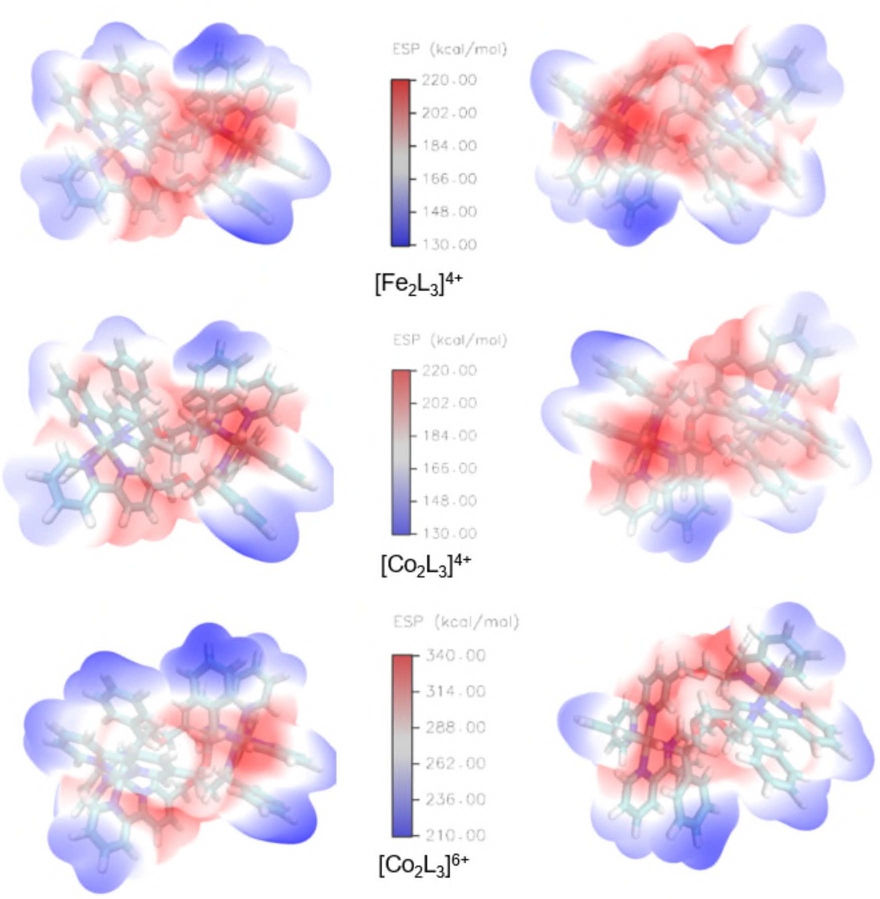

Figure S15. the molecular electrostatic potential (ESP) map of the optimised structures of the metallohelices.

5.3 Computational Procedure for Spatial Analysis of hydrophobicity

The hydrophobicity calculations were achieved using ORCA program. The hydrophobicity index was defined by the principle that the solvent molecule was found near the hydrophilic region of the solute and far from the hydrophobic region of the solute. The previously optimised structures of metallohelices were used as basis to perform the calculation.

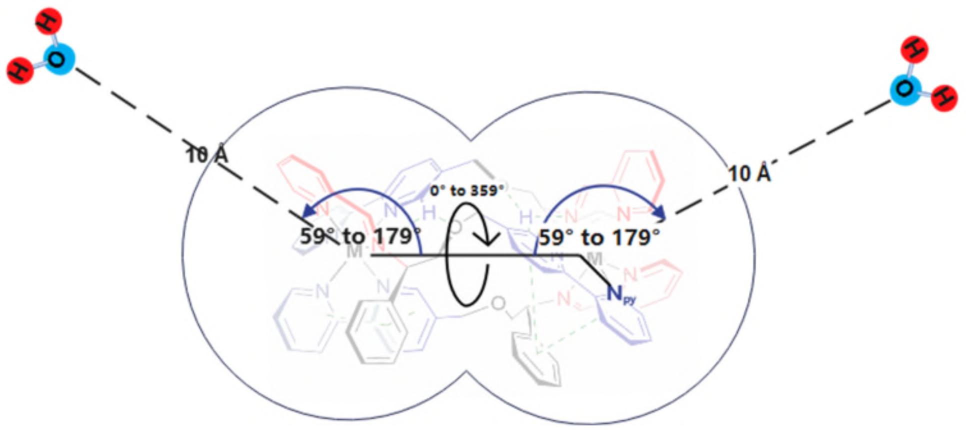

Figure S16. Schematic diagram of the hydrophobicity calculation, where the polar coordinates describing the watering oxygen atom's spatial relationship with metallohelices

i. Coordinate System Setup

A polar coordinate system was implemented to describe the positional relationship between the oxygen atom in a water molecule and the static atom configurations of the bimetallic helicate. The coordinate system was defined with one of the metal atoms serving as the origin, the primary axis formed by the two metal atoms, and a coordinating bipyridyl nitrogen atom (Npy) acting as a reference.

ii. Angular and Radial Variation:

The spatial coordinates of the water oxygen atom were systematically defined through a fixed azimuthal angle relative to nitrogen atom Npy (varying from 0° to 359° in 5° increments, 72 azimuthal angles) and a fixed polar angle (ranging from 179° to 59° in 10° increments, 13 polar angles). The radial distance from the origin (initially set at 10 Å) was allowed to vary freely, encapsulating the degrees of freedom within the system.

iii. Energy Minimization

An energy minimization was conducted using the PM7 basis set. The minimization process involved the variation of the water oxygen position, azimuthal and radial angles, and the positions of water hydrogen atoms. All other atoms within the metallohelicate were frozen at fixed positions.

iv. Calculations

Two sets of calculations were performed: the first with the origin at one metal centre, resulting in 936 distinct calculations, and the second with the origin at the other metal centre, thus covering the "sphere" of helicate with an additional 936 calculations.

v. Coordinate Conversion and Visualization:

Polar coordinates of the water oxygen atoms from each of the remaining 1868 calculations were converted into Cartesian coordinate system. Subsequently, these coordinates were plotted in MATLAB2019 using the scatter3 function. The obtained plots were fused with the electron density map of the molecule thereby demonstrating the hydrophobicity of the molecule. The colour scale of the resulting plot represented the minimized energy levels, ranging from relative hydrophilic (blue) to hydrophobic (red).

Table S1. The result of the hydrophobicity calculation of three metallohelices, which contains the hydrophobicity map (top image refers to front view, bottom image refers to back view, coloured according to the energy scale, hydrophilic (blue) to hydrophobic (red)), the upper and lower limits of the energy.

Bimetallic Complexes	Hydrophilicity plots	Lower Kcal/mol	Upper Kcal/mol
**Fe(II)**	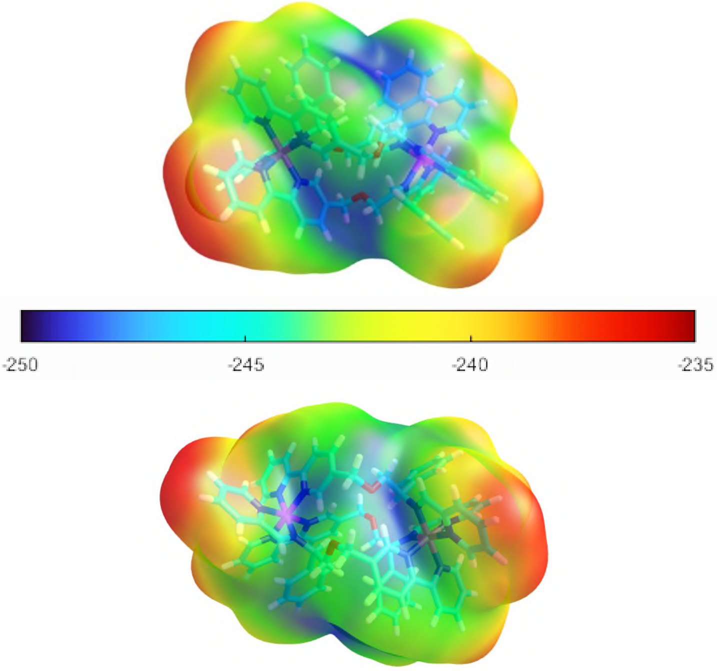	-250.95	-221.67

**Co(II)**	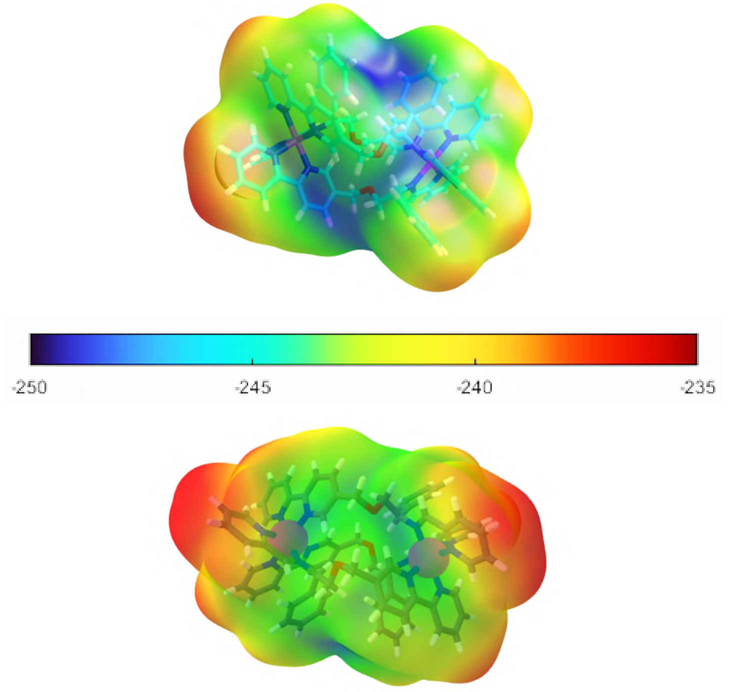	-246.58	-234.34
**Co(III)**	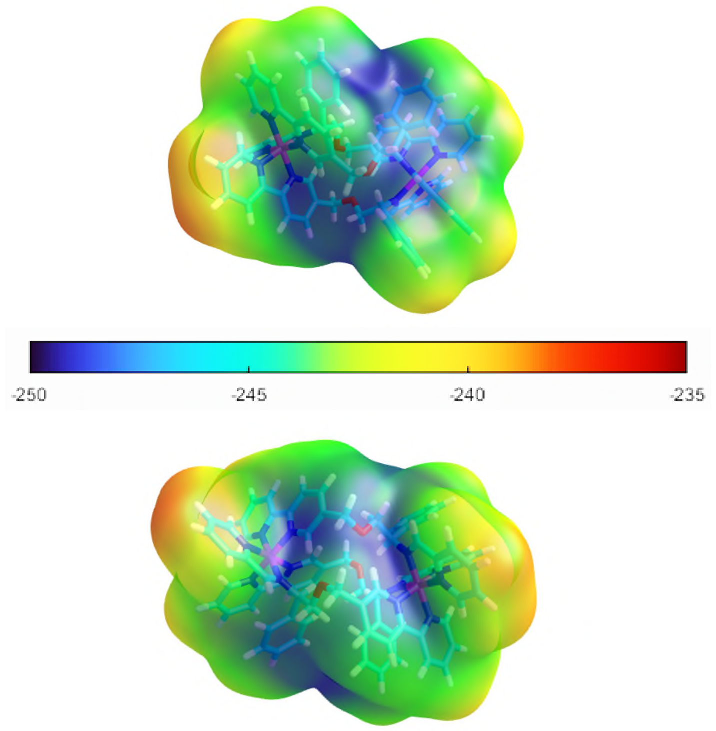	-253.19	-238.78

5.4 Input example

```
! RI B3LYP def2-SVP def2/J D3BJ TIGHTSCF Opt Grid3 FinalGrid5 slowconv
%basis newgto Co "def2-TZVP" end end
%maxcore  2048
%pal nprocs   1 end
%scf MaxIter 1500 end
%cpcm
smd true
SMDsolvent "water"
end
* xyz   4   7
N     20.96000000    7.46100000    2.62900000
C     19.87200000    7.61800000    1.89700000
O     25.06100000    8.35400000    5.57800000
N     22.97600000    6.81100000    4.30400000
C     18.77700000    8.40200000    2.26700000
N     29.21400000    8.06500000    6.57900000
C     18.97300000    9.12800000    3.42500000
O     28.49800000    5.00000000    2.39500000
N     31.54800000    6.95400000    7.21500000
C     20.08400000    8.96500000    4.21500000
N     32.71800000    7.40600000    4.24200000
C     21.02300000    8.08700000    3.80100000
O     27.38100000    9.71700000    2.97600000
N     30.36300000    6.02300000    4.40800000
C     22.18000000    7.70900000    4.64400000
N     24.19800000    4.65900000    2.20400000
C     23.99400000    6.28400000    5.16900000
C     24.50100000    7.24000000    6.27200000
N     21.62400000    4.34500000    2.89100000
N     31.59700000    9.95500000    5.89200000
C     23.58500000    4.97000000    5.81700000
N     30.12500000    9.15800000    3.76600000
C     24.42300000    3.88800000    5.85200000
N     23.89000000    7.69000000    1.45900000
C     23.96600000    2.67100000    6.42200000
N     22.21600000    6.04400000    0.15600000
C     22.71300000    2.58900000    6.92800000
C     21.86200000    3.61500000    6.96400000
C     22.30000000    4.88900000    6.37500000
C     25.69200000    9.31200000    6.50400000
C     28.09400000    8.63700000    6.19200000
C     26.93000000    8.75100000    7.02000000
C     27.05700000    8.22500000    8.34300000
C     28.25700000    7.59200000    8.70800000
C     29.30900000    7.53200000    7.81200000
C     30.61900000    6.90700000    8.13000000
C     30.82200000    6.17300000    9.26900000
C     32.06500000    5.55600000    9.50700000
C     33.05300000    5.66500000    8.53700000
C     32.76000000    6.38800000    7.39400000
C     33.84100000    8.10500000    4.12100000
C     34.86900000    7.71300000    3.25900000
C     34.69300000    6.52200000    2.46900000
C     33.49900000    5.85900000    2.58900000
C     32.54700000    6.28200000    3.46500000
```

C	31.23100000	5.57800000	3.59400000
C	29.09800000	5.31300000	4.70400000
C	28.72200000	4.27600000	3.62500000
C	29.19000000	4.55100000	6.03200000
C	28.15300000	4.67700000	6.94400000
C	28.22400000	3.99300000	8.13200000
C	29.27600000	3.17800000	8.41900000
C	30.32400000	3.04300000	7.52900000
C	30.30300000	3.77900000	6.30100000
C	27.80700000	4.17300000	1.41200000
C	25.44100000	4.85700000	1.75000000
C	26.43400000	3.85100000	1.77900000
C	26.06700000	2.60400000	2.30600000
C	24.75600000	2.40200000	2.72300000
C	23.83000000	3.43900000	2.67800000
C	22.41300000	3.29300000	3.06100000
C	21.90900000	2.13200000	3.63900000
C	20.61200000	2.05900000	4.00600000
C	19.81800000	3.17900000	3.89900000
C	20.37600000	4.30300000	3.29200000
C	32.35900000	10.30700000	6.93800000
C	32.63200000	11.66900000	7.15800000
C	32.08000000	12.64100000	6.33200000
C	31.33900000	12.24100000	5.27400000
C	31.05400000	10.87000000	5.07800000
C	30.27700000	10.40200000	3.97300000
C	29.46800000	8.62700000	2.56500000
C	28.40900000	9.52800000	1.95500000
C	30.56500000	8.31200000	1.53200000
C	30.44800000	7.15700000	0.70700000
C	31.44700000	6.82800000	-0.20200000
C	32.59600000	7.60600000	-0.28000000
C	32.71300000	8.73400000	0.50700000
C	31.72100000	9.10000000	1.37500000
C	26.26300000	10.44600000	2.49800000
C	24.65100000	8.51500000	2.15100000
C	25.35300000	9.61000000	1.57900000
C	25.16800000	9.83200000	0.24700000
C	24.35300000	8.98600000	-0.50000000
C	23.75600000	7.89600000	0.11100000
C	22.90900000	6.91200000	-0.60700000
C	22.88700000	6.81500000	-2.00600000
C	22.11200000	5.87100000	-2.62900000
C	21.36900000	5.00300000	-1.82400000
C	21.45400000	5.07700000	-0.47600000
H	19.83300000	7.16700000	1.06100000
H	18.31600000	9.76400000	3.68500000
H	20.18900000	9.45300000	5.02400000
H	22.31900000	8.16400000	5.46700000
H	24.78400000	6.08000000	4.58700000
H	23.75300000	7.53100000	6.85100000
H	25.18600000	6.79600000	6.83300000
H	25.30200000	3.95100000	5.49800000
H	24.53800000	1.91300000	6.45200000
H	22.42200000	1.75400000	7.27500000
H	21.00200000	3.52700000	7.35600000
H	21.72700000	5.64500000	6.37200000

H	25.88400000	10.16000000	6.02800000
H	25.07600000	9.50800000	7.25300000
H	28.05100000	8.99500000	5.31200000
H	26.34600000	8.30400000	8.96400000
H	28.34700000	7.20400000	9.57100000
H	30.12000000	6.07800000	9.90400000
H	32.22700000	5.07700000	10.31200000
H	33.90500000	5.25900000	8.65500000
H	33.42500000	6.48800000	6.72200000
H	33.94600000	8.89700000	4.63500000
H	35.37700000	6.20700000	1.89000000
H	33.33100000	5.09400000	2.05100000
H	31.04900000	4.80600000	3.07200000
H	28.36400000	5.98700000	4.77100000
H	29.45700000	3.62200000	3.50600000
H	27.90200000	3.78800000	3.88800000
H	27.40200000	5.22800000	6.74800000
H	27.52400000	4.09000000	8.76800000
H	29.28900000	2.69900000	9.24000000
H	31.05200000	2.46600000	7.72900000
H	31.02700000	3.73900000	5.69200000
H	27.80300000	4.64700000	0.54100000
H	28.31000000	3.32900000	1.29200000
H	25.66200000	5.71000000	1.39500000
H	26.70700000	1.90500000	2.37600000
H	24.49200000	1.54800000	3.04500000
H	22.48000000	1.38500000	3.77500000
H	20.24900000	1.24400000	4.33500000
H	18.92600000	3.18600000	4.22600000
H	19.83400000	5.07100000	3.16300000
H	32.70500000	9.64500000	7.52300000
H	32.22000000	13.56400000	6.50600000
H	31.00600000	12.88400000	4.66000000
H	29.86600000	11.03000000	3.39000000
H	29.02600000	7.76500000	2.81400000
H	28.80300000	10.39900000	1.69700000
H	28.01800000	9.10700000	1.14800000
H	29.67700000	6.60600000	0.77800000
H	31.34500000	6.07200000	-0.76900000
H	33.29800000	7.36200000	-0.87200000
H	33.49400000	9.26700000	0.44300000
H	31.81200000	9.89900000	1.88200000
H	26.58700000	11.23800000	2.00000000
H	25.73300000	10.76700000	3.27000000
H	24.73600000	8.37000000	3.08700000
H	25.60100000	10.56700000	-0.17400000
H	24.20200000	9.15800000	-1.42300000
H	23.41400000	7.40900000	-2.52900000
H	22.07800000	5.81000000	-3.57600000
H	20.80400000	4.35500000	-2.22700000
H	20.97800000	4.44500000	0.05100000
Co	22.63800000	6.13700000	2.23900000
Co	30.96000000	7.91900000	5.35000000
H	18.00083672	8.45596183	1.68155113
H	33.23088188	11.86758075	8.02217948
H	35.64666664	8.29679457	3.20889823

*

6. Redox properties of the cobalt triplex metallohelices

Electrochemistry

Cyclic voltammetry (CV) experiments were conducted using a three-electrode configuration and an Ivium compactstat (Ivium Technologies B.V., Netherlands). A saturated calomel electrode (SCE, CH Instruments, IJ Cambria Scientific Ltd., UK) and a coiled platinum wire (Goodfellow, UK) served as the reference and counter electrodes, respectively. A gold disk electrode of 2 mm diameter (CH Instruments) was used as the working electrode. The SCE was routinely monitored for any drift, relative to a master SCE reference electrode and the coiled Pt wire counter electrode was flame cleaned before use to remove any unwanted contaminants. Prior to use the working electrode was mechanically polished first using a micropolish alumina (0.05 µm) paste (Buehler, Germany) on a microcloth pad (Buehler, Germany), and then polished on a wetted (with ultra-pure water) alumina-free pad and thoroughly rinsed. The experiment was performed using a 1 mM aqueous solution of Λ-**7** complex containing 0.1 M KCl under argon, to remove dissolved oxygen from solution. The pH of the solution was 6.42.

CVs were recorded from -0.3 V *vs* SCE to +0.6 V *vs* SCE, continuously for 10 scans. The first CV scan of the Λ-**7** complex, shows a clear oxidation peak (Co(II) to Co(III)) at 0.235 V *vs* SCE (0.482 V *vs* NHE) and a reduction peak (Co(III) to Co(II)) at 0.142 V *vs* SCE (0.389 V *vs* NHE). $E_{1/2}$ of the Λ-**7** redox reaction is 0.436 V *vs* NHE (reference electrode corrected) with a peak-to-peak separation, ΔE_p, of 93 mV.

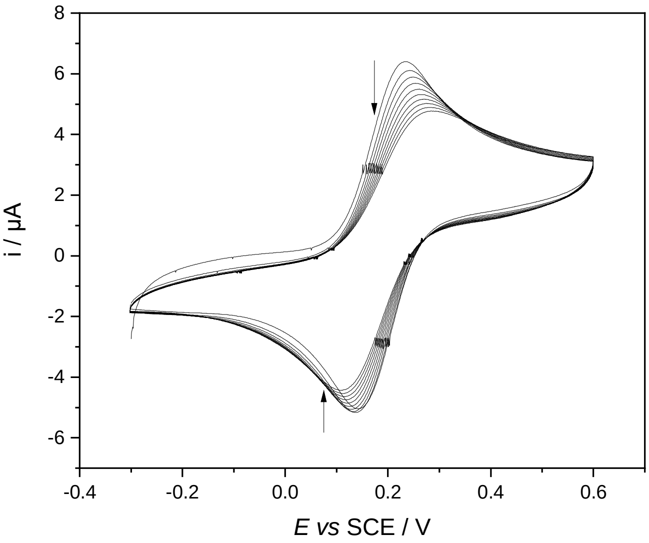

Figure S17. Co(II) complex oxidation: 1 mM Λ-7 in 0.1 M KCl, using a gold disk electrode (2 mm diameter), 100 mV/s (scanning from -0.3 to 0.6 V *vs* SCE) First scan peak: oxidation peak at 0.235 V, 6.40 μA; reduction peak at 0.142 V, -5.04 μA.

UV spectral analysis of Co(III) reduction

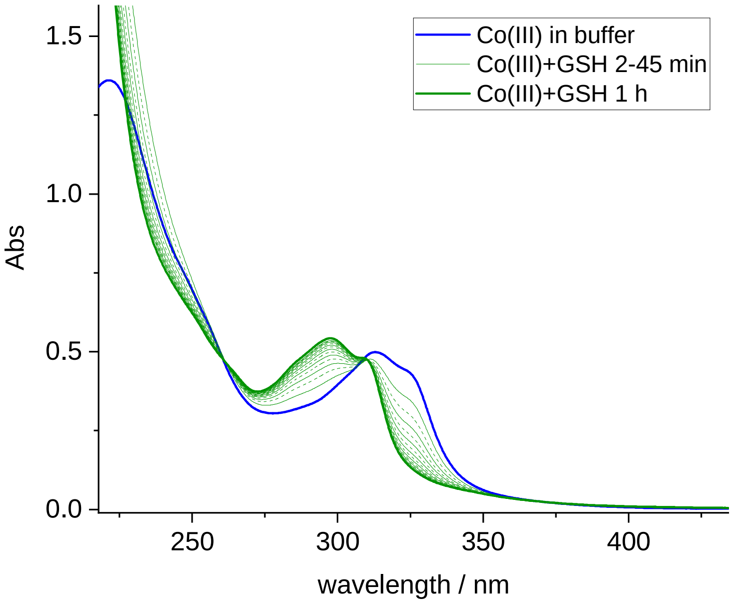

Figure S18. Time course UV-Vis spectra of S_c,Λ_{Co},HHT-[Co_2**L**$_3$]Cl_6 (**Λ-6**) (10 μM) following the addition of glutathione (1 mM) in Tris buffer (pH 7.4)

^{1}H NMR analysis

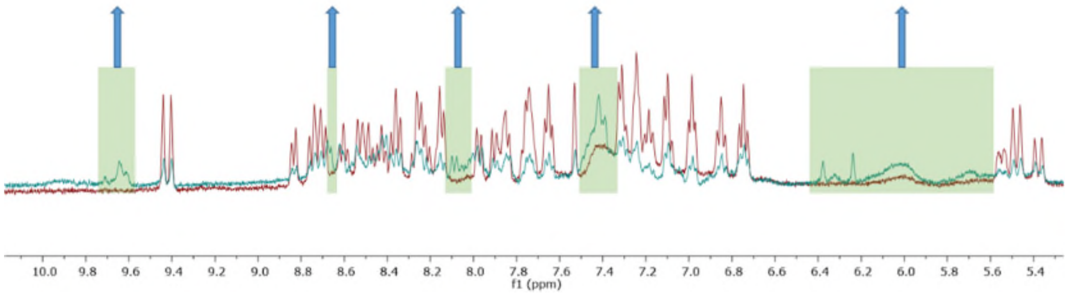

Figure S19. ^{1}H NMR spectra (D_2O, 298 K, 300 MHz) of 2 mM pure S_c,Λ_{Co},HHT-[Co_2**L**$_3$]Cl_6 (**Λ-6**) (red line) in 10 mM PBS buffer and 2 mM S_c,Λ_{Co},HHT-[Co_2**L**$_3$]Cl_6 (**Λ-6**) with 10 mM GSH (cyan line) in 10 mM PBS buffer.

For the mixture of Co(III) complex and GSH, the peaks of Co(III) complex were reduced by factor of 2.4 and there were new peaks (in pale green region) observed.

7. Cancer cell studies

Cell culture and Chemosensitivity

ARPE-19 human retinal epithelial cells (non-cancer) were obtained from ATCC and cultured in DMEM/F12 culture medium containing l-glutamine (2.5mM), sodium pyruvate (0.5mM), HEPES buffer (15mM) and foetal calf serum (10% v/v). HCT116 human colorectal carcinoma cells[8] were grown in DMEM containing L-glutamine (2mM) and foetal calf serum (10% v/v). All cell lines were routinely maintained as monolayer cultures and sub-cultured or harvested for chemosensitivity studies when approximately 70-80% confluent. Cells were seeded into 96-well tissue culture plates at a density of 2×10^3 cells/well for HCT116, and ARPE-19 cells/well. Plates containing cells were incubated for 24 h at 37°C in an atmosphere of 5% CO_2 prior to drug exposure. Cell media (200 µl) was added to the control cells and differing concentrations (0 to 50 µM) of drug solution (200 µl) were added to the remaining wells. All complexes were directly dissolved in cell media. The plates were incubated for a further 72 or 96 h at 37°C in an atmosphere of 5% CO_2. Media was removed and replaced with fresh media prior to the assay. 3- (4,5-Dimethylthiazol-1-yl)-2,5-diphenyltetrazolium bromide (MTT) solution (0.5 mg/ml, 20µl per well) was added to each well and incubated for a further 4 h at 37°C in an atmosphere of 5% CO_2. Upon completion, all solutions were removed from the wells and dimethyl sulfoxide (150 µl,) was added to each well to dissolve the purple formazan crystals. A Thermo Scientific Multiskan EX microplate photometer was used to measure the absorbance at 540 nm. Lanes containing 100% cell media and untreated cells were used as a blank and 100% cell survival respectively. Cell survival was determined as the true absorbance of treated cells divided by the true absorbance of untreated controls; this value was expressed as a percentage. The IC_{50} values were determined from a plot of percentage cell survival against drug concentration (µM). All assays were conducted in triplicate and the mean IC_{50} ± standard deviation was determined.

Table S2. Cell viability (IC_{50}) of the triplex metallohelices, measured by MTT assay in HCT116 and ARPE-19. Experiments were performed in triplicate, cells were incubated with compounds for 96 h, results are expressed as mean values ±SD.

		IC_{50} Value (μM)±SD	
	**Metal ion**	**HCT116**	**ARPE-19**
$CoCl_2$	Co(II)	>91.23	>100
Λ-**5**	Co(II)	0.28 ± 0.05	0.86 ± 0.24
Δ-**5**	Co(II)	2.44 ± 0.24	6.0 ±1.5
Λ-**6**	Co(III)	0.31 ± 0.04	0.7 ± 0.4
Δ-**6**	Co(III)	4.22 ± 1.70	7.1 ± 1.3
Λ-$\mathbf{Fe^{II}}$	Fe(II)	1.42 ± 0.39	10.0 ± 1.8
Δ- $\mathbf{Fe^{II}}$	Fe(II)	21.4 ± 1.4	31 ± 12

Cell culture and Chemosensitivity – manuscript Table 1

Cells of human cervical carcinoma (HeLa) were kindly provided by Professor B. Keppler, University of Vienna (Austria), human colorectal carcinoma (HCT116) were kindly provided by Dr. M. Brazdova, Institute of Biophysics, Brno (Czech Republic), human breast cancer (MCF7), human rhabdomyosarcoma (RD), human pancreatic cancer (PSN1), human colon cancer (Colo320) and normal human lung tissue (MRC5 pd30) were purchased from the European Collection of Authenticated Cell Cultures (ECACC) (Salisbury, UK). RD and PSN1 cells were cultured in RPMI 1640 medium (PAA, Pasching, Austria) supplemented with gentamycin (50 μg.mL-1, Serva, Heidelberg, Germany) and 10% heat inactivated fetal bovine serum (PAA). The remaining cell lines were grown in DMEM medium (high glucose, 4.5 g.L-1, PAA) supplemented with gentamycin (50 μg.mL-1, Serva, Heidelberg, Germany) and 10% heat inactivated fetal bovine serum (PAA). Medium for MRC5-pd30 was further supplemented with 1% non-essential amino acids (Sigma-Aldrich, Prague, Czech Republic). If not stated otherwise, the cells were cultured as adherent monolayers in a humidified incubator (37 °C, 5% CO_2) and subcultured twice or thrice a week.

Antiproliferative activities of the metallohelices were determined using the assay based on the tetrazolium compound MTT [3-(4,5-dimethyl2-thiazolyl)-2,5-diphenyl-2H-tetrazolium bromide]. The cells were seeded at optimal pre-determined densities in 96-well plates,

incubated overnight and treated with a range of concentrations of the tested compounds for 72 hours. MTT was added (20 µL of 1.25 mg/mL PBS solution) for the final 3 hours. The medium was then replaced with 100 µL DMSO to dissolve the formazan products of cellular metabolization of MTT. Absorbance was read at 570 nM (reference 620 nm) and IC_{50} values were calculated.

Cellular uptake

HCT116 cells (as in manuscript Table 2) were seeded at a density of $2x10^6$ cells/Petri dish and grown overnight. Culture medium was replaced with fresh medium containing 5 µM metallohelices (5 mL) and the cells were incubated for 8 hours. The cells were then harvested (trypsinization), washed (PBS), counted and pelleted by centrifugation. It was verified that at the time of harvest, at least 95% of cells was viable as determined by the trypan blue exclusion test. Cell pellets were digested using the microwave acid digestion system (CEM Mars®). Cobalt amounts were determined with ICP-MS.

Cell death

To investigate the mode of cell death induced in HCT116 cells by the tested compounds annexin-V/propidium iodide assay was employed. The cells were seeded at a density of $3x10^5$ cells per 60 mm Petri dish, grown overnight and treated with the tested compounds at roughly equitoxic concentrations corresponding to $3xIC_{50(72\ h)}$ values for 48 hours. Cell treated with staurosporine (2 µM, 4 h) and ethanol (10%, 1h) were included as positive controls for apoptosis and necrosis, respectively. The cells were then harvested, washed with PBS and incubated with annexin-V (Pacific Blue conjugate, 5 µL/100 µL) and propidium iodide (1 $µg.mL^{-1}$) for 15 min. Cells were analyzed by flow cytometry (BD FACS Verse) and processed with FCS Express 6 software (DeNovo; Glendale, CA).

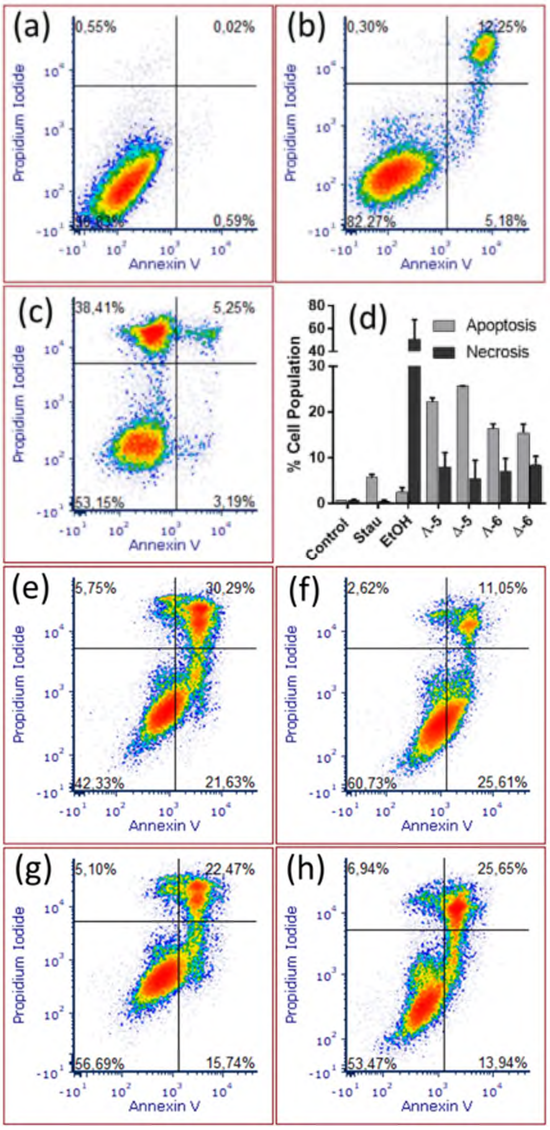

Figure S20. Cell death determined in HCT116 cells following the treatment with Co metallohelices using Annexin V/Propidium iodide staining. Density plots: bottom left quadrant – live cells, bottom right – early apoptotic cells, upper left – necrotic cells, and upper right – dead or late apoptotic cells. (a) Non-treated control; (b) Staurosporine; (c) Ethanol; (d) Apoptotic and necrotic populations; (e) Λ-**5;** (f) Δ-**5**; (g) Λ-**6** and (h) Δ-**6**. The experiment was performed twice, and 3×10^4 cells were analysed in each sample.

Cell cycle

HCT116 cells were seeded in 60 mm Petri dishes ($3x10^5$ cells/well) and allowed to attach for 16 hours. Culture medium was replaced with fresh medium containing the studied compounds at concentrations corresponding to 1x-, 2x- and $3xIC_{50}$ values (determined with MTT after 72

hours) and the cells were incubated for another 24 hours. The cells were collected, washed with PBS and resuspended in Annexin-V binding buffer (100 µL) containing 5 µL Pacific Blue Annexin-V and 1 µg propidium iodide. After 15 minutes staining, the samples were analyzed with BD FACSVerse flow cytometer.

Real-time cell growth monitoring

HCT116 cells were seeded in 96-well E-plates (Roche; $1x10^3$ cells/well) and grown and monitored with Real-time cell analyzer (RTCA) (XCelligence RTCA SP Instrument, Roche) for 24 hours. Various concentrations of the investigated agents were added to the wells. The concentrations were chosen to induce similar cell growth inhibition as determined by the MTT assay. The impedance of individual wells was recorded for another four days. Cell index (CI), an arbitrary unit reflecting overall status of the cells (count, morphology, adhesion) was obtained. CI was normalized by dividing CI at a time point by CI at the time of compounds addition (24 hours).

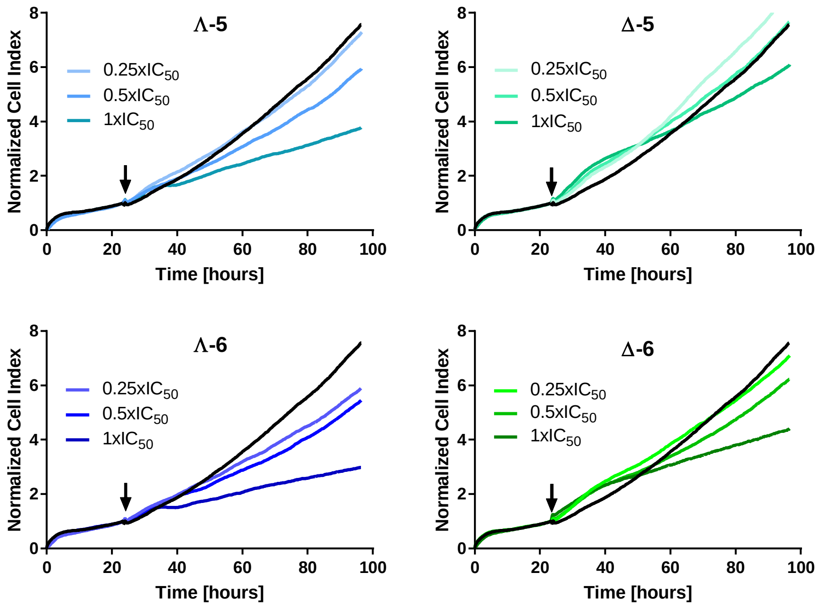

Figure S21. Real-time impedance-based cell growth monitoring. TCRP profiles of HCT116 cells treated with increasing concentrations of the tested compounds. Black line – untreated control. The metallohelices concentrations were chosen to induce similar cell growth inhibition and are based on IC_{50} values determined with MTT assay after a 72-hour treatment. Cell indices were normalized to the values at the time of compound addition (arrow).

Nuclear uptake

HCT116 cells were seeded in Petri dishes at a density of $2x10^6$ cells/dish and allowed to attach overnight. The cells were then exposed to 5 µM compounds for 8 hours. The cells were then harvested by trypsinization and processed with Nuclei EZ Prep Kit according to the recommended protocol. The nuclei were then counted and pelleted. The pellets were digested with the microwave acid (HCl) digestion system (CEM Mars®). Cobalt amounts were determined with ICP-MS.

DNA transcriptional activity

This assay is based on the use of a fluorescent analog of UTP (UTP-gamma-AmNS; Jena Bioscience, Jena, Germany) as one of the nucleotide substrates.[9] Incorporation of UMP in RNA strand by RNA polymerase leads to the release of gamma-AmNS, which exhibits higher intrinsic fluorescence than UTP-gamma-AmNS. Circular pBR322 plasmid DNA was used as a template at the concentration of 0.96 µg/100 µl. Transcription was performed in a total volume of 28 µl in 10 mM Tris-HCl buffer (pH 7.6), 100 mM KCl, 5 mM $MgCl_2$, 0.1 mM ATP, CTP and GTP, 0.01 mM UTP-gamma-AmNS, 2 mM DTT, and increasing concentrations of metallohelices. After 10 min of pre-incubation at RT, one unit of *E. coli* RNA polymerase holoenzyme (New England Biolabs, Beverly, MA, USA) was added to each sample and incubated for 2 h at 37 ºC. RNA polymerization was stopped by addition of 42 µl of 50 mM EDTA and transcription products were detected by measuring the sample fluorescence intensity using Varian Cary Eclipse spectrofluorophotometer with the following parameters: excitation wavelength 330 nm, emission wavelength 463 nm, excitation and emission slit widths 10 nm, and integration time 3 s.

Topoisomerase I-catalyzed relaxation of negatively supercoiled DNA

Metellohelices at various concentrations were mixed with 0.2 µg of supercoiled pUC19 plasmid DNA (2686 bp; New England Biolabs, Beverly, MA, USA) in 35 mM Tris–HCl, pH 8.0, 72 mM KCl, 5 mM $MgCl_2$, and 5 mM DTT in a total volume of 8 µl and left for 15 min at RT. 1 unit of DNA topoisomerase I (Takara Bio Inc., Shiga, Japan) was then added into each

reaction mixture and incubated for 1 hour at 37 ºC. Reactions were terminated by the addition of 0.5 µl of 10% SDS. All samples were then mixed with 1.5 µL of loading dye (0.25% bromophenol blue, 60% glycerol) and loaded onto a 1% agarose gel running at RT with 1×TAE (Tris–acetate/EDTA) buffer and the voltage set at 45 V. The gels were then stained with GelRed (Millipore, USA) and photographed on a UV transilluminator.

Confocal microscopy of H2AX phosphorylation

HCT116 cells were seeded on coverslips in 6-well plates at a density of 1.5x10^5 cells/well and grown overnight. The cells were treated with the compounds at 4 µM concentration for 24 hours. The cells were then washed with PBS, fixed with 4% para-formaldehyde, permeabilized with PBST (0.1% Triton X-100 in PBS), blocked with 5% FBS and immunostained with primary (anti-γH2AX antibody; abcam; ab81299) and secondary (Goat Anti-Rabbit IgG-Alexa Fluor® 488; abcam; ab150077) antibodies and mounted with ProLong Diamond Antifade with DAPI (Invitrogen). The imaging was performed with Leica TCS SP8 SMD (Leica microsystems GmbH, Wetzlar, Germany). The images were sequentially scanned with first scan 405 nm (diode laser)/emission window 420 to 550 nm, second scan 488 nm (white laser)/emission window 520 to 600 nm). Pinhole was set to 1 AU.

Flow cytometry analysis of H2AX phosphorylation

HCT116 cells were seeded in 60 mm Petri dishes at a density of 3x10^5 cells/well and grown overnight. The cells were then treated with the compounds at a series of concentrations (0.5 µM, 1 µM, 2 µM and 4 µM for 24 hours. The cells were then fixed with 4% para-formaldehyde and stained with Alexa Fluor 488 conjugated Anti-phospho Histone H2AX antibody (16-202A, Merck Millipore). The cells were analyzed by flow cytometry (BD FACSVerse) and the data were analyzed using FCS Express software (DeNovo software; Glendale, CA).

Tubulin polymerization

Tubulin polymerization in the absence and presence of the investigated metallohelices was assessed with Tubulin Polymerization Assay, Fluorescence Based (Cytoskeleton, Inc.) following the manufacturer´s instructions. Briefly, 2 mg/mL (final concentration) tubulin in

tubulin polymerization buffer (80 mM PIPES pH 6.9, 2.0 mM $MgCl_2$, 0.5 mM EGTA and 15% glycerol) and variable concentrations of metallohelices were added in 96-well plate (white, flat bottom). GTP was added to final concentration of 1 mM immediately before inserting the plate in fluorimeter. SPARK reader (Tecan) was set to 37 °C; Ex/Em=360/420 nm. Tubulin polymerization was recorded as increase of fluorescence.

Microtubule imaging

HCT116 cells were seeded on coverslips in 6-well plates at a density of $1.5x10^5$ cells/well and grown overnight. The cells were treated with the compounds at concentrations corresponding to 5x- and 10xIC_{50} for 6 hours. The cells were then washed with PBS, fixed with 4% para-formaldehyde, permeabilized with PBST (0.1% Triton X-100 in PBS), blocked with 5% FBS and immunostained with primary (anti-α-Tubulin antibody; abcam; ab176560) and secondary (Goat Anti-Rabbit IgG-Alexa Fluor® 488; abcam; ab150077) antibodies and mounted with ProLong Diamond Antifade with DAPI (Invitrogen). The imaging was performed with Leica TCS SP8 SMD (Leica microsystems GmbH, Wetzlar, Germany). The images were sequentially scanned with first scan 405 nm (diode laser)/emission window 420 to 550 nm, second scan 488 nm (white laser)/emission window 520 to 600 nm). Pinhole was set to 1 AU.

Actin polymerization

Actin Polymerization Biochem Kit™ (Cytoskeleton, Inc.) was used to determine the effect of the tested compounds on actin polymerization in vitro. The assay uses pyrene conjugated G-actin and enhanced fluorescence is recorded when F-actin is formed. The assay was performed according to the recommended protocol. Pyrene actin (0.4 mg mL^{-1}) was polymerized in actin polymerization buffer in microplate format at room temperature on SPARK™ multimode reader (Tecan) (Ex. 360 nm and Em. 410 nm).

Actin imaging

HCT116 cells were grown, treated and fixed as in the case of microtubule imaging and stained with Alexa FluorTM 488 Phalloidin (Thermofisher Scientific, 40 min). The cells were mounted

with ProLongTM Diamond Antifade with DAPI (Invitrogen). The imaging was performed with Leica TCS SP8 SMD microscope at the same setting as for microtubule imaging.

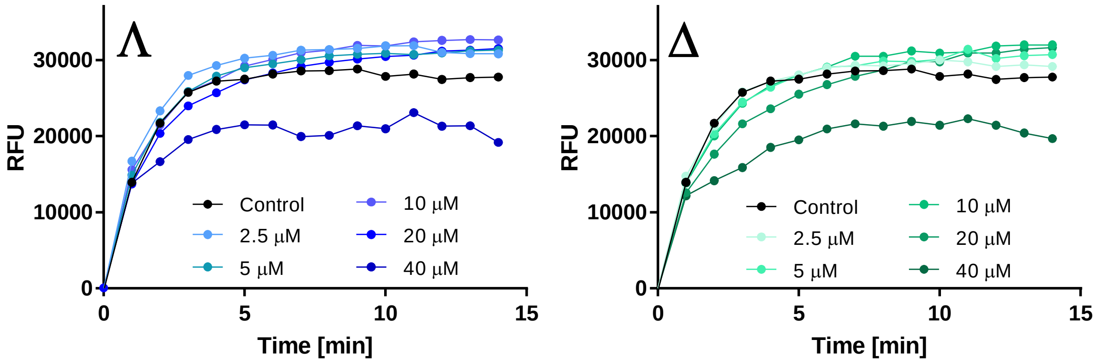

Figure S22. Actin polymerization. Fluorescence growth (Ex/Em=360/410 nm) reflecting pyrene conjugated actin polymerization was recorded in the absence (black) and in the presence of growing concentrations of Co(II)-Λ (blue) (**Λ-5**) and Co(II)-Δ (green) (**Δ-5**) at room temperature.

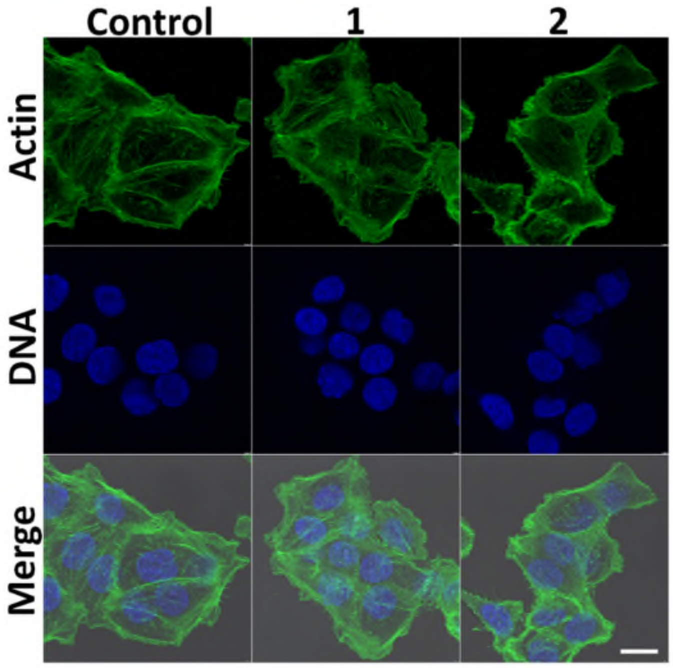

Figure S23. Confocal fluorescence images - Actin. HCT116 cells were non-treated (Control) or treated with Co(II) triplex metallohelices at concentrations corresponding to 10xIC$_{50}$ **(1,2)** for 6 hours. Actin filaments were stained with Alexa Fluor® 488 conjugated phalloidin. C9ell nuclei were counterstained with DAPI. **(1)** Λ**-5**, **(2)** Δ**-5**. Scale bar represents 20 μm.

8. References

1. A. D. Faulkner, R. A. Kaner, Q. M. Abdallah, G. Clarkson, D. J. Fox, P. Gurnani, S. E. Howson, R. M. Phillips, D. I. Roper, D. H. Simpson and P. Scott, *Nat. Chem.*, 2014, **6**, 797-803.
2. F. Neese, *Rev.: Comput. Mol. Sci*, 2012, **2**, 73-78.
3. F. Weigend and R. Ahlrichs, *Phys. Chem. Chem. Phys.*, 2005, **7**, 3297-3305.
4. S. Grimme, S. Ehrlich and L. Goerigk, *J. Comput. Chem.* , 2011, **32**, 1456-1465.
5. S. Grimme, J. Antony, S. Ehrlich and H. Krieg, *J. Chem. Phys.*, 2010, **132**.
6. T. Lu and F. Chen, *J. Comput. Chem.*, 2013, **117**, 3100-3108.
7. W. Humphrey, A. Dalke and K. Schulten, *J. Mol. Graphics*, 1996, **14**, 33-38.
8. F. Bunz, P. M. Hwang, C. Torrance, T. Waldman, Y. Zhang, L. Dillehay, J. Williams, C. Lengauer, K. W. Kinzler and B. Vogelstein, *J. Clin. Investig.*, 1999, **104**, 263-269.
9. F. Luckel, K. Kubo, K. Tsumoto and K. Yoshikawa, *FEBS Lett.*, 2005, **579**, 5119-5122.
